# Species diversity patterns in managed Scots pine stands in ancient forest sites

**DOI:** 10.1371/journal.pone.0219620

**Published:** 2019-07-11

**Authors:** Ewa Stefańska-Krzaczek, Monika Staniaszek-Kik, Katarzyna Szczepańska, Tomasz H. Szymura

**Affiliations:** 1 Botanical Garden, University of Wrocław, Wrocław, Poland; 2 Department of Geobotany and Plant Ecology, Faculty of Biology and Environmental Protection, University of Lodz, Lodz, Poland; 3 Department of Botany and Plant Ecology, Wroclaw University of Environmental and Life Sciences, Wrocław, Poland; 4 Department of Ecology, Biogeochemistry and Environmental Protection, University of Wrocław, Wrocław, Poland; Technical University in Zvolen, SLOVAKIA

## Abstract

Continuity in forest habitats is crucial for species diversity and richness. Ancient Scots pine forests are usually under forest management, which disturbs vegetation and causes differentiation in terms of tree stand age. To date, vegetation variability in ancient Scots pine forests has not been examined based on tree stand age classes. In the present study the continuity of a large Scots pine forest complex was investigated, and a system of sampling plots established in five tree stand age classes: initiation stands (4–10 years), young stands (20–35 years), middle-aged stands (45–60 years), pre-mature stands (70–85 years) and mature stands (95–110 years). Species composition, including vascular plants, bryophytes and lichens, on soil, tree trunks, and coarse woody debris, was analyzed. Based on existing classifications systems, forest species and ancient forest species groups were distinguished. In the studied ancient Scots pine forests the species pool and richness were relatively low, and the vegetation consisted mostly of generalist species. Cryptogams, which can grow on diverse substrates, were the most abundant species. Moreover, most species could tolerate both forest and non-forest conditions. Age class forests provided different environmental niches for species. Initiation stands were optimal for terrestrial light-demanding species, and in terms of species composition, initiation stands were most specific. Young stands were most preferred by species on coarse woody debris, and at this stage of stand maturation epiphytic species re-appeared. The oldest stands were not rich in forest specialists, i.e. species of closed forest and ancient forest species. Cryptogams of closed forests inhabited different substrates, and they were not associated only with the oldest stands. The low number of forest specialists in the oldest stands may be a general feature of acidophilus pine forests. However, it may also be a result of the lack of species sources in the vicinity of maturing pine stands. In managed forests a frequent diversity pattern is an increase in a species pool and richness after clear-cut logging. In the present study we obtained higher species pools in initiation and young stands, but richness was similar in all tree stand age classes. This resulted from taking into account species of different substrates (terrestrial, epixylous and epiphytic species) which changed their participation in the vegetation of subsequent stages of tree stand development.

## Introduction

In Europe, *Pinus sylvestris* is one of the most abundant tree species [1,2]. Since it has great economic importance, its widespread distribution across forest sites is attributed more to its commercial value than its ecological competence [2,3,4]. However, in Central Europe, Scots pine is a natural dominant tree species in forests where broadleaved trees are not able to grow [5], e.g. on acid sandy soils [6]. Based on the phytosociological approach, those Scots pine forests belong to the *Dicrano-Pinion* alliance [5,7,8].

Scots pine stands in mineral soils are usually exposed to regular intense human activity. As a result, forest floor vegetation is periodically disturbed and the biodiversity is greatly influenced by forest management activities [9–14]. Nevertheless, in lowlands, where most productive sites have been converted into arable fields, intensively managed Scots pine forests may be the only remnants of ancient forests [15]. The prolonged habitat continuity is beneficial to forest plant specialists referred to as ancient forest species [16,17]. However, intensive forest management may diminish the availability of microsites and substrates [11,18], while promoting non-forest species [19–25]. Therefore, the continuity of forest habitats may not ensure high quality habitat conditions that are optimal for forest specialists [26–28]. Previous studies have demonstrated that the characteristics of vegetation of ancient Scots pine forests differ from those of recent forests on arable lands [15,29–31]. Nevertheless, managed Scots pine forests, even in habitats with long-term continuity, represent age class forests, which may be differentiated based on variation in tree stand age. Stand age classes differ based on management intensity, tree stand structure, and forest floor species richness and composition [19,20,32]. The most considerable silvicultural practice in commercial pine stands is clear-cutting [33]. Stand removal changes forest microclimate. Moreover, clear-cutting is followed by site preparation and replanting. Vegetation characteristics of young stands are different from those of previous forest vegetation, and they represent non-forest stages in stand rotation periods [19,20,34–36]. Crown closure in young tree stands is only a stage when the forest microclimate starts to develop and shade-tolerant species may find favourable conditions once more [37,38]. Subsequently, thinning is carried out in each decade until logging time [33], which disturbs understory vegetation [13,39,40].

Therefore, ancient and managed Scots pine forests, even within a single forest complex with the same history of land use, may differ in species composition because of the spatial mosaics of tree stands in different age classes. The dynamics of the understory under pressure of forest management activities have been studied extensively [41,42]. However, further studies are still required since a combination of the technical attributes of the management practices, tree species composition, and forest site types, including its continuity, may influence the diversity and composition of an understory [16,35,43,44].

To date, vegetation variability in ancient Scots pine forests based on stand age differentiation has not been examined. Examination of such differentiation could be crucial in future assessments of differences in biodiversity between ancient and recent forests. In addition, deeper insights into such age-related diversity could facilitate the formulation of better forest management strategies, which could in turn benefit biodiversity conservation. In the present study, we examined forest floor vegetation along stand age gradients in managed Scots pine forest on sites with habitat continuity (ancient forest sites). We posed the following questions: 1) Do species number and cover vary along gradients of tree stand age on all available substrates? 2) How do vegetation structure and composition change along a tree stand age gradient? 3) How are forest indicator species and ancient forest species distributed along a tree stand age gradient?

## Materials and methods

### Preselection of sampling plots

**Fig 1:**
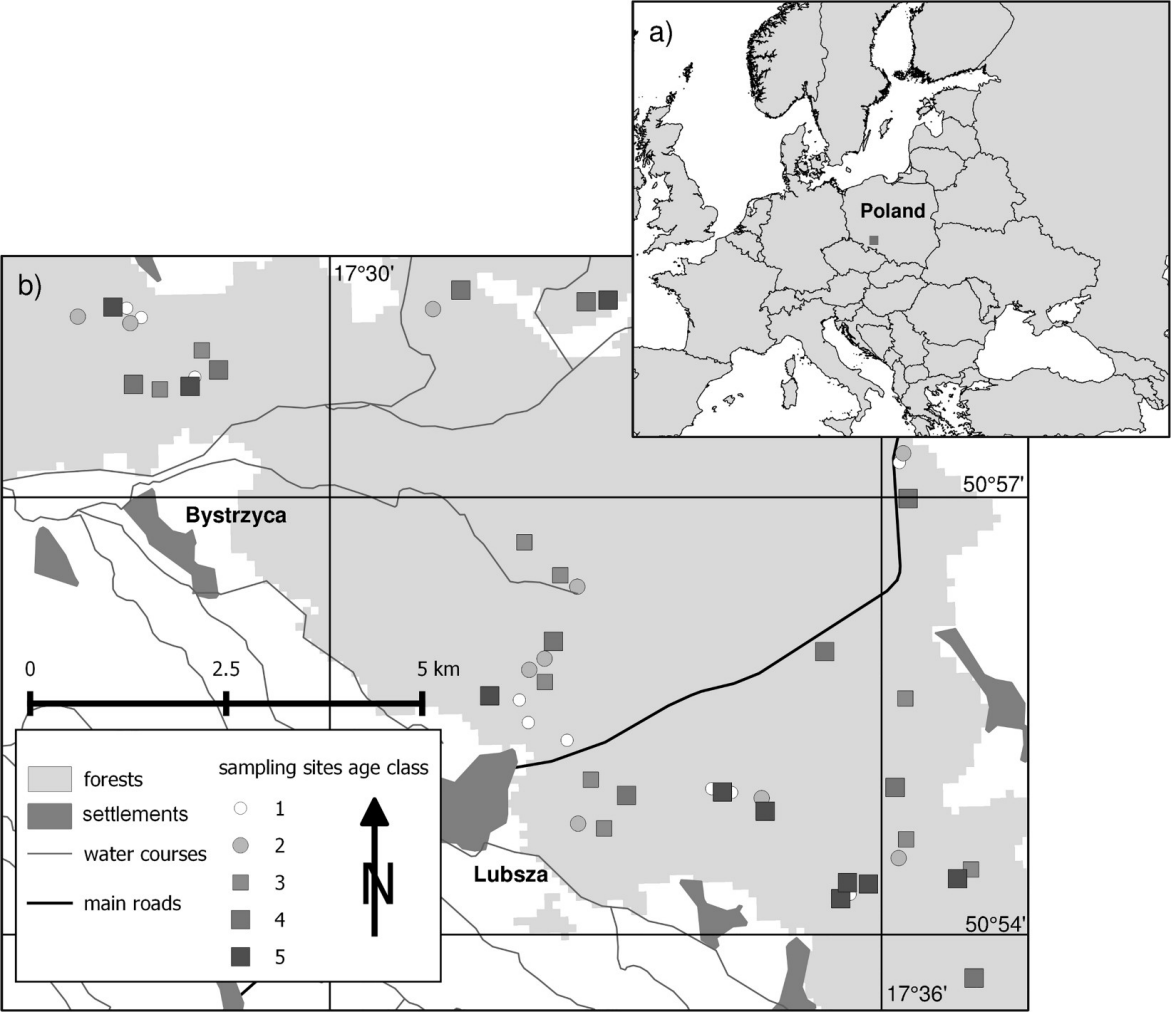
**Location of the study area in Poland (a) and distribution of sampling plots (b).** Stand age classes: 1 –initiation stands (4–10 years), 2 –young stands (20–35 years), 3 –middle-aged stands (45–60 years), 4 –pre-mature stands (70–85 years), 5 –mature stands (95–110 years).

The study was carried out in a part of a large forest complex, “Bory Stobrawskie”, in Opole Silesia, Poland, Central Europe (centre of the area 50°56'33.35"N, 17°32'51.39"E) (Fig 1A). We studied plots in the Brzeg Forest District (Permit number 5/15, Brzeg Forest District of the Regional Directorate of State Forest in Katowice). We first compared historical maps from different periods: Kriegskarte von Schlesienn 1748, Ur-Messtischblatt 1824, Messtischblatt 1880s and 1930s, and recent topographical maps. Detailed descriptions of the historical maps and their analyses can be found in Szymura et al. [45,46]. A comparison of the maps revealed that most of the area in Bory Stobrawskie was continuously forested since as early as 1748. Therefore, we considered the forest complex as an ancient woodland. The studied forests have been managed intensively through clear-cutting. New tree generations are planted after site preparation (removal of logs and ploughing), and stand thinning is carried out every 10 years. Shrub layers emerge in pre-mature and mature stands spontaneously or are planted. The rotation period in the studied forest district is 90–110 years.

Based on the State Forest Database (System Informatyczny Lasów Państwowych SILP) and our analysis of historical and contemporary maps, we selected study areas according to the following criteria: they should be 1) pure single-layer Scots pine stands, and 3) ancient forests on 3) albic brunic arenosols (Dystric) with moderate soil moisture, according to the World Reference Base for Soil Resources [47]. Within areas that met the criteria, we chose forest sections in five age classes: class 1 (4–10 years), initiation stands before crown closure; class 2 (20–35 years), young stands exhibiting intensive height growth; class 3 (45–60 years), middle-aged stands after the culmination of height growth; class 4 (70–85 years), pre-mature stands exhibiting increasing biomass; and class 5 (95–110 years), mature stands before logging. In forest management, one age class represents 20 years of tree stand age. We used similar classes; however, for each age class in the present study, we chose tree stands representing only part of age ranges. In this way, we attempted to exclude forest communities with species composition exhibiting transition between age classes. Subsequently, we selected ten forest sections for each age class randomly, based on their dispersion across the study area (Fig 1B).

### Fieldwork

We established sampling plots in the middle of the selected forest sections, where vegetation was visually representative of the sections, while avoiding atypical elements, such as canopy gaps and footpaths. Within the plots (200 m^2^, radius 8 m), we recorded data on basic characteristics of the vegetation structure. We delimited six community layers: high tree layer (T1, higher than 10 m), low tree layer (T2, 8.1–10 m), high shrub layer (S1, 3.1–8 m), low shrub layer (S2, 0.6–3 m), herb layer (H, up to 0.5 m, including herbs, dwarf-shrubs, ferns, and seedlings of tree species), and moss layer (M, including liverworts, mosses, and lichens). We visually estimated the percentage cover of layers (T1, T2, S1, S2, H, M) and the percentage cover of coarse woody debris (CWD). In addition, diameters at breast height (1.3 m above ground level) of ten trees closest to the centre of the plot, and the height of one tree of average diameter at breast height were measured (Table 1). Diameters of trees were not measured in the initiation stands.

**Table 1:** General characteristic of community structure in tree stand age classes.

	Class 1		Class 2		Class 3		Class 4		Class 5		*H*	*p*
	Mean	SD	Mean	SD	Mean	SD	Mean	SD	Mean	SD		
DBH [mm]	-	-	156.65	12.25	225.35	25.05	281.85	25.77	298.77	22.76	44.75	0.0000
Stand height (T1) [m]	1.80	0.42	15.80	3.39	23.30	2.36	27.00	3.46	27.10	2.85	40.29	0.0000
Cover T1 [%]	0.00	0.00	67.00	6.75	57.50	7.91	51.50	7.84	46.50	7.09	37.57	0.0000
Cover T2 [%]	0.50	1.58	0.00	0.00	3.10	5.32	10.00	11.79	15.50	11.65	25.44	0.0000
Cover S1 [%]	0.50	1.58	4.50	7.98	4.60	4.88	15.10	7.96	19.00	12.87	25.78	0.0000
Cover S2 [%]	64.50	14.99	7.30	6.41	10.30	7.02	7.10	4.04	6.20	3.61	25.7	0.0000
Cover H (%)	44.50	15.17	32.50	11.37	66.50	10.81	63.50	9.14	68.00	14.57	29.18	0.0000
Cover M (%)	24.50	15.54	68.50	13.75	77.50	10.34	76.00	14.87	67.50	13.59	25.78	0.0000
Cover CWD (%)	12.00	5.87	24.50	9.85	8.50	2.42	9.50	3.69	9.00	4.59	21.44	0.0003

Stand age classes: 1 –initiation stands (4–10 years), 2 –young stands (20–35 years), 3 –middle-aged stands (45–60 years), 4 –pre-mature stands (70–85 years), 5 –mature stands (95–110 years); *H*, *p*–values of statistics for Kruskal-Wallis test; DBH–diameter at breast height (not measured in class 1); layers: T1 –high tree layer, T2 –low tree layer, S1 –high shrub layer, S2 –low shrub layer, H–herb layer, M–moss layer; CWD–coarse woody debris; for detailed definition of layers see the section Fieldwork.

Four sampling squares of 4 m^2^ (2 × 2 m) were established 4 m from the centres of the plots at directions of 45°, 135°, 225°, and 315°. We used such small plots instead of standard forest relevé (100–400 m^2^) to facilitate the examination of the occurrence of cryptogam species on all available substrates (soil, CWD, and living tree trunks). All species were recorded in all layers (T1, T2, S1, S2, H, M) in the squares. In addition, the cover of each species in each layer was estimated using the following scale: 0.1, 0.5, 1, 5, 10, 15, 20, 25, 30, 35, 40, 45, 50, 55, 60, 65, 70, 75, 80, 85, 90, 95, 100%. The scale was also used for estimating the amount of CWD, including branches, small logs, and snags. Within each square, the presence of bryophyte and lichen species on CWD and living tree trunks up to a height of 50 cm was noted, but their cover was not assessed (presence/absence data). If a species occurred on different types of substrates within a square (soil, CWD, tree trunks), it was recorded separately for each substrate. In total, we examined 50 plots and 200 squares (10 plots and 40 squares for each tree age class; S1 Database).

### Data analysis

For the study area and for each age class, the total number of species (species pool) was calculated. In the case of bryophytes and lichens, we also calculated the species pool and percentage of species occurring on one, two, and three substrate types (soil, CWD, and tree trunks). For further analysis we combined data from four squares in one sampling plot. For each sampling plot we calculated the number of all species (species richness). Then we calculated species richness and cover in T–M layers, and species richness on CWD and tree trunks. In addition, for each sampling plot, we calculated indices for light, moisture, reaction and nitrogen: LEIV, FEIV, REIV, NEIV [48]. We assigned indicator values to each of the species, and in each sampling plot we calculated mean unweighted LEIV, FEIV, REIV, NEIV. Indicator values for species were obtained from Schmidt et al. [49] and Hill et al. [50].

Spearman's rank correlation coefficients were computed between all variables calculated for the sampling plots and stand age. Mean values of all variables were also calculated for each stand age class. Significance of differences between age classes was tested using the Kruskal-Wallis test, and multiple comparisons of average ranks as post-hoc tests. We used p = 0.05. All the calculations were performed in the STATISTICA program [51].

Species composition was examined using ordinations. In this way, we aimed to determine general trends in species composition, demonstrate floristic similarities or dissimilarities between classes, and determine the distribution of species groups in age classes. Prior to the analysis, we omitted species with a single occurrence in the entire dataset. Cover values obtained after the combination of square data were transformed into an ordinal scale ranging from 1 to 11: 0.1–0.5→1; 0.55–1→2; 1.5–10→3; 10.5–20→4; 20.5–30→5; 30.5–40→6; 40.5–50→7; 50.5–60→8; 60.5–70→9; 70.5–80→10; 80.5–90→11. For species on CWD and tree trunks, for which cover was not assessed, a minimum value of one was assumed. We also excluded tree species recorded in T–S layers from the species list to pay more attention to distribution of species that were independent from silvicultural practices. The mean cover of tree species in T and S layers were included in the analysis as supplementary variables. As supplementary variables, we also used CWD cover, LEIV, FEIV, REIV, and NEIV. The gradient length was 3.1 SD, so that we could apply detrended correspondence analysis (DCA). The ordination was performed in CANOCO 5 [52].

Variability in species composition in age classes was evaluated by comparing fidelity (a species concentration in vegetation units), frequency, and cover of species, in the JUICE program [53]. We used phi coefficient as a fidelity measure [53]. Only positive values were taken into account, with significance assessed by the Fisher exact test at p = 0.01. For each stand age class we determined: diagnostic species (phi>20), constant species (frequency >50%), and dominant species (cover >20% on a minimum of 20% of the plots representing the age class).

To determine the distribution of forest and ancient forest species along a tree stand age gradient, we calculated for each plot richness and cover of forest species (cover only in the case of species in H and M layers) according to Schmidt et al. [49], and the richness and cover of ancient forest species (AFS), defined by Hermy et al. [17]. We also analyzed changes in cover for each of the ancient forest species along a tree stand age gradient. The responses of species were obtained based on a generalized linear model in CANOCO [52]. Besides, we assessed whether recorded AFS revealed a tendency to co-occur, especially in particular age classes. We combined all AFS into one group of species and then by JUICE [53] we checked the number of plots were the AFS group was present. The minimum number of species from the group required to determine the presence of the group in a plot was at least half the number of species from the group [54].

The nomenclature of vascular plants was according to Euro+Med PlantBase [55], whereas bryophyte nomenclature was according to Ochyra et al. [56], with the exception of *Rosulabryum moravicum* (Podp.) Ochyra et Stebel [57]. In addition, the nomenclature of liverworts was according to Klama [58]. Lichen naming was based on Index Fungorum [59] and MycoBank Database [60].

## Results

### Species number and cover

**Fig 2:**
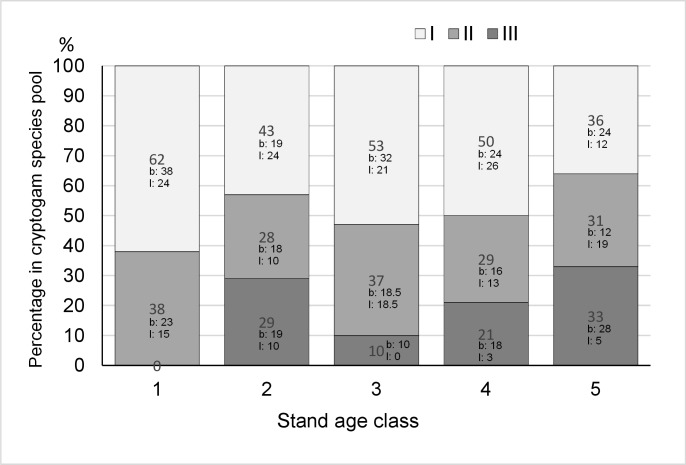
Proportion of cryptogam species observed on one (I), two (II) and three (III) substrates (soil, coarse woody debris and trunks) dividing in lichens (l) and bryophytes (b).

The total number of species (species pool) was 116 in the studied area. In the successive stand age classes the species pool was as follows: initiation stands: 72, young stands: 72, middle-aged stands: 60, pre-mature stands: 62, mature stands: 60. The average number (species richness) per plot was 25 (26, 27, 23, 22, and 25 in the successive stand age classes, differences not significant). Lichens and bryophytes were the most abundant species and accounted for 65% of the species pool (65%, 71%, 63%, 61%, and 70% in age classes). A considerable proportion of the species pool was represented by species occurring in two or three substrates (Fig 2).

The highest positive correlations with stand age were observed for species richness and cover in T2, S1, and species cover in H and M layers (particularly with cover of terrestrial bryophytes) and with the species richness of all epiphytes. Conversely, the strongest negative correlations with stand age were observed for richness and cover of terrestrial lichens (in M layer) and richness of lichens on CWD (S1 Table).

Initiation stands (age class 1) differed significantly from the other age classes based on the higher richness of terrestrial cryptogams and non-tree vascular species. The initiation stands were also distinct based on the higher richness and cover of terrestrial lichens, and the lower average cover of terrestrial bryophytes (S1 Table). In the young stands (class 2), the richness and cover of terrestrial lichens decreased, while the average cover of terrestrial bryophytes and the species richness on CWD, i.e. bryophytes, increased. In addition, in the young stands, epiphytes were recorded for the first time (S1 Table). In classes 3–5 there were no significant changes in species richness and cover in particular layers and on specific substrates. Moreover, the richness of all species was similar in age classes (differences were not significant).

### Vegetation structure and species composition

**Fig 3:**
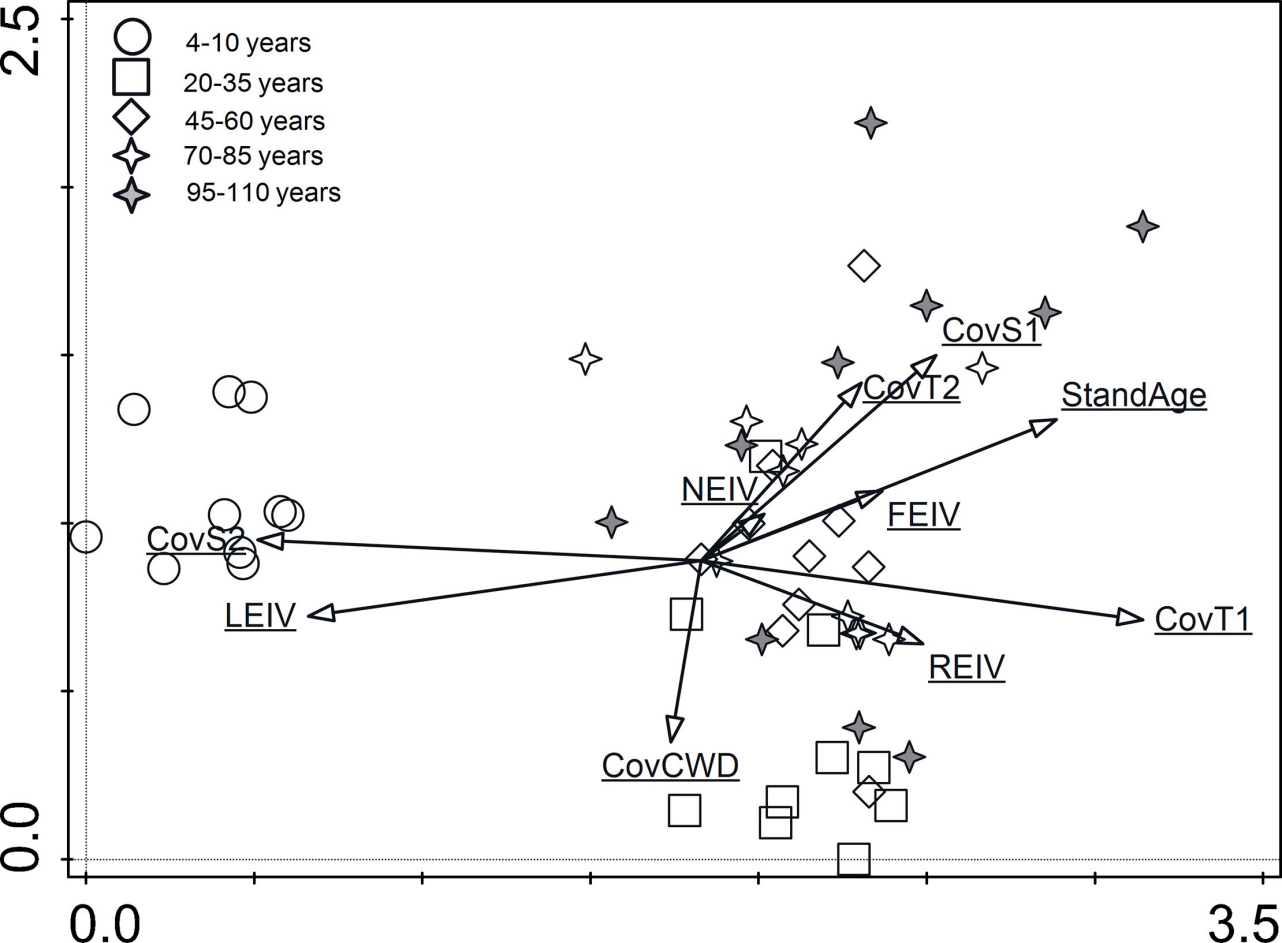
Ordination plot of floristic relations between stand age classes. Supplementary variables: CovT1 –cover of high tree layer, CovT2 –cover of low tree layer, CovS1 –cover of high shrub layer, CovS2 –cover of low shrub layer; CovCWD–cover of coarse woody debris, LEIV–light index, FEIV–moisture index, REIV–reaction index, NEIV–nitrogen index, based on ecological indicator values for species.

The eigenvalue of the first axis of the unconstrained ordination analysis (DCA) was 0.4073, and it explained 9.97% of the variability in the data set. The first axis separated the initiation stands clearly, and the representative points formed a group on the left side of the diagram (Fig 3). Stand age classes 2–5 were similar with regard to species composition and formed an extensive group of representative points on the right side of the diagram (Fig 3). Three species were common (constant) in all stand age classes: *Avenella flexuosa*, *Vaccinium myrtillus*, and *Pleurozium schreberi* (Table 2, S2 Table). *A*. *flexuosa* was dominant in the initiation stands while *V*. *myrtillus* and *P*. *schreberi* were dominant in age classes 2–5.

**Table 2:** Species composition of vegetation in stand age classes.

Stand age class	Diagnostic species (fidelity measured by phi coefficient multiplied by 100 ≥20) Constant species (frequency ≥50%) Dominant species (cover ≥20% on at least 20% of studied plots)
1. Initiation stands (4–10 years)	Diagnostic species: *Betula pendula* [S2] 69.0, *Pinus sylvestris* [S2] 94.1; *Betula pendula* [H] 57.9, **Calamagrostis epigeios [H] 57.9, **Calluna vulgaris [H] 59.0, **Carex pilulifera [H] 46.8, **Juncus effusus [H] 50.5; *Campylopus introflexus [M] 59.0, *Ceratodon purpuraeus [M] 80.7, **Cladonia chlorophaea [M] 50.5, **Cladonia macilenta [M] 59.0, **Dicranella heteromalla [M] 61.6, **Placynthiella icmalea [M] 87.5, *Placynthiella oligotropha [M] 93.7, **Pohlia nutans [M] 63.9, *Polytrichum juniperinum [M] 58.5; **Placynthiella icmalea [CWD] 57.9, *Trapeliopsis flexuosa [CWD] 59.0 Constant species: **Avenella flexuosa [H] 100, *Quercus petraea* [H] 60, *Rubus* sp. [H] 50, **Vaccinium myrtillus [H] 100, **Dicranum polysetum [M] 50, **Hypnum jutlandicum [M] 60, **Orthodicranum montanum [M] 60, **Pleurozium schreberi [M] 80, **Polytrichastrum formosum [M] 80, **Cladonia coniocraea [CWD] 70, **Orthodicranum montanum [CWD] 50, **Pohlia nutans [CWD] 70 Dominant species: *Pinus sylvestris* [S2] 100; **Avenella flexuosa [H] 40
2. Young stands (20–35 years)	Diagnostic species: ***Herzogiella seligeri [CWD] 43.6, **Hypnum cupressiforme [CWD] 40.7; **Cladonia chlorophaea [Pinus] 50.0, **Cladonia coniocraea [Pinus] 42.1, ***Coenogonium pineti [Pinus] 66.7, **Hypnum jutlandicum [Pinus] 50.5, ***Lophocolea heterophylla [Pinus] 48.0, **Micarea micrococca [Pinus] 51.4, **Orthodicranum montanum [Pinus] 48.6 Constant species: *Pinus sylvestris* [T1] 100; *Picea abies* [S2] 60; **Avenella flexuosa [H] 100, *Quercus petraea* [H] 60, **Vaccinium myrtillus [H] 100; **Dicranum polysetum [M] 70, **Dicranum scoparium [M] 50, **Pleurozium schreberi [M] 100, **Pseudoscleropodium purum [M] 70, **Sciuro-hypnum oedipodium [M] 80; **Brachythecium rutabulum [CWD] 60, **Cladonia coniocraea [CWD] 60, ***Coenogonium pineti [CWD] 60, ***Lophocolea heterophylla [CWD] 90, **Orthodicranum montanum [CWD] 80, **Placynthiella dasaea [CWD] 80, **Plagiothecium curvifolium [CWD] 50, **Pohlia nutans [CWD] 60, **Sciuro-hypnum oedipodium [CWD] 70; *Cladonia* sp. [Pinus] 50 Dominant species: *Pinus sylvestris* [T1] 100; **Avenella flexuosa [H] 20, **Vaccinium myrtillus [H] 30; **Dicranum polysetum [M] 20, **Pleurozium schreberi [M] 70
3. Middle-aged stands (45–60 years)	Diagnostic species: *Lecanora conizaeoides [Pinus] 46.4, *Lepraria* sp. [Pinus] 40.7 Constant species: *Pinus sylvestris* [T1] 100; *Sorbus aucuparia* [S2] 50; **Avenella flexuosa [H] 100, *Pinus sylvestris* [H] 50, *Quercus petraea* [H] 60, **Vaccinium myrtillus [H] 100, **Vaccinium vitis-idaea [H] 70; **Pleurozium schreberi [M] 100, **Polytrichastrum formosum [M] 50, **Pseudoscleropodium purum [M] 80, **Sciuro-hypnum oedipodium [M] 80; **Cladonia coniocraea [CWD] 50, ***Lophocolea heterophylla [CWD] 80, **Orthodicranum montanum [CWD] 70, **Pleurozium schreberi [CWD] 60, **Sciuro-hypnum oedipodium [CWD] 60; **Hypocenomyce scalaris [Pinus] 50, ***Lophocolea heterophylla [Pinus] 70 Dominant species: *Pinus sylvestris* [T1] 100; **Vaccinium myrtillus [H] 100; **Pleurozium schreberi [M] 80, **Pseudoscleropodium purum [M] 20
4. Pre-mature stands (70–85 years)	Diagnostic species: *Picea abies* [S1] 65.6; **Dryopteris carthusiana [H] 50.0; **Cladonia digitata [Pinus] 62.5 Constant species: *Pinus sylvestris* [T1] 100; *Fagus sylvatica* [S1] 50, ***Calamagrostis arundinacea [H] 50, **Avenella flexuosa [H] 90, *Pinus sylvestris* [H] 50, *Quercus petraea* [H] 80, **Vaccinium myrtillus [H] 100, **Vaccinium vitis-idaea [H] 60; **Plagiothecium curvifolium [M] 50, **Pleurozium schreberi [M] 100, **Pseudoscleropodium purum [M] 50, **Sciuro-hypnum oedipodium [M] 60; ***Lophocolea heterophylla [CWD] 70, **Pleurozium schreberi [CWD] 80, **Pohlia nutans [CWD] 50; **Hypocenomyce scalaris [Pinus] 60, *Lepraria* sp. [Pinus] 50, ***Lophocolea heterophylla [Pinus] 50 Dominant species: *Pinus sylvestris* [T1] 100; *Quercus petraea* [T2] 20; **Vaccinium myrtillus [H] 100; **Pleurozium schreberi [M] 70, **Pseudoscleropodium purum [M] 30
5. Mature stands (95–110 years)	Diagnostic species: *Quercus petraea* [S1] 65.6; **Hylocomium splendens [M] 54.7; ***Chaenotheca ferruginea [Pinus] 50.5; ***Lophocolea heterophylla [Quercus] 51.9 Constant species: *Pinus sylvestris* [T1] 100; *Quercus petraea* [T2] 50; *Quercus petraea* [S2] 70; **Avenella flexuosa [H] 70, *Quercus petraea* [H] 70, **Vaccinium myrtillus [H] 100, **Vaccinium vitis-idaea [H] 60; **Brachythecium rutabulum [M] 50, **Dicranum polysetum [M] 50, **Plagiothecium curvifolium [M] 50, **Pleurozium schreberi [M] 100, **Pohlia nutans [M] 50, **Polytrichastrum formosum [M] 60, **Pseudoscleropodium purum [M] 80, **Sciuro-hypnum oedipodium [M] 80; ***Lophocolea heterophylla [CWD] 60, **Orthodicranum montanum [CWD] 60, **Placynthiella dasaea [CWD] 70, **Pleurozium schreberi [CWD] 60; **Hypocenomyce scalaris [Pinus] 60 Dominant species: *Pinus sylvestris* [T1] 100; *Quercus petraea* [T2] 30; *Quercus petraea* [S1] 30; **Vaccinium myrtillus [H] 100; **Pleurozium schreberi [M] 90, **Pseudoscleropodium purum [M] 20

***species name–species 1.1: restricted to closed forests

**species name–species 2.1: occurring in forests and in open land

*species name–species 2.2: occurring in forests, but preferring open land; underlined diagnostic species are also constant species; layer/substrate in brackets: T1 –high tree layer, T2 –low tree layer, S1 –high shrub layer, S2 –low shrub layer, H–herb layer, M–bryophyte-lichen layer, CWD–coarse woody debris, Pinus, Quercus, Picea–trunks as substrates for epiphytes.

**Fig 4:**
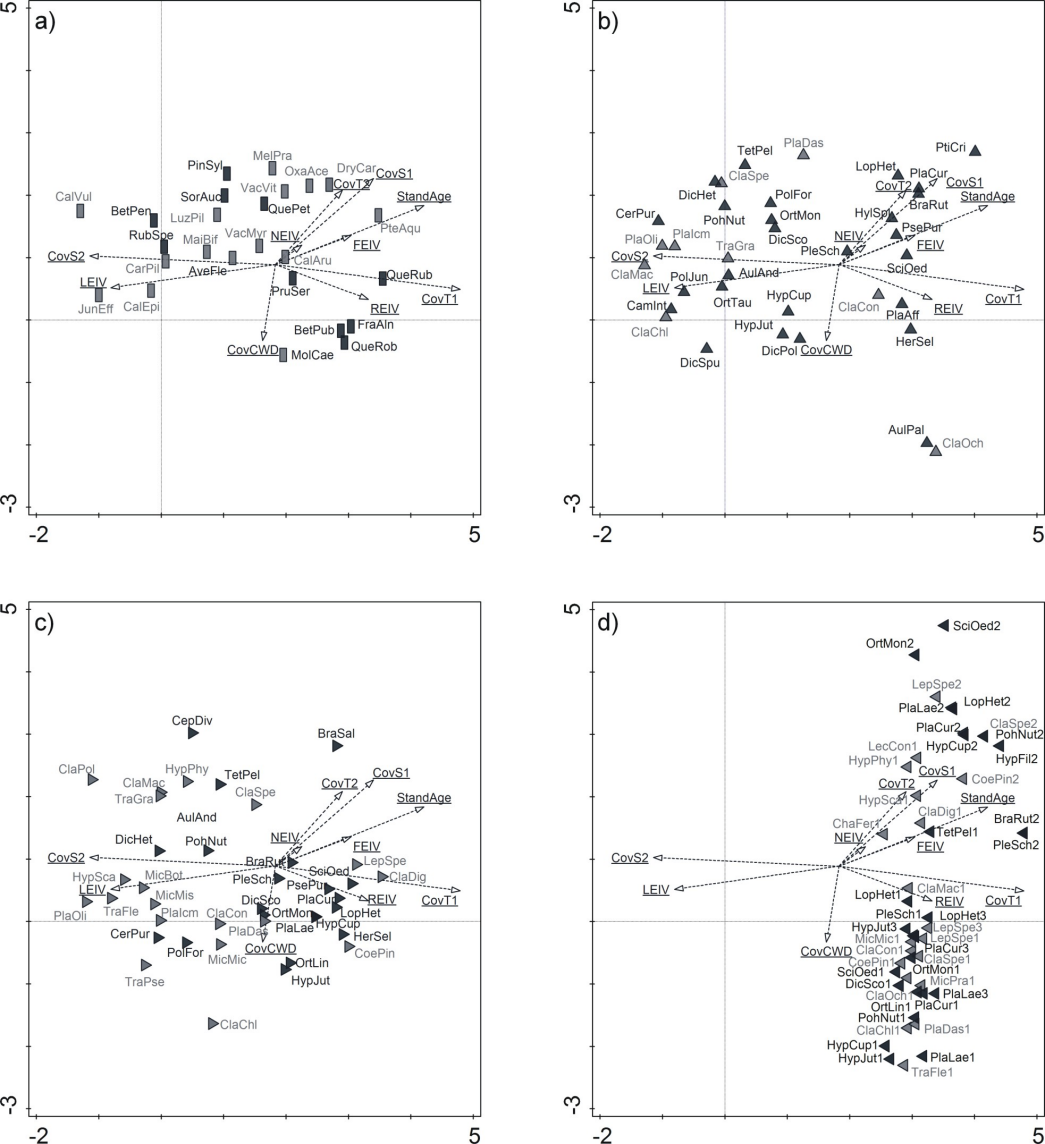
**Distribution of species in the studied forests a) vascular plants, b) terrestrial lichens and bryophytes, c) lichens and bryophytes on coarse woody debris, d) lichens and bryophytes on tree trunks.** a) black boxes: trees and shrubs of herb layer, grey boxes: non-tree vascular plants; b-d) black triangles: bryophytes, grey triangles: lichens; d) numbers after species codes–trunks as substrates for epiphytes: 1 –Pinus, 2 –Quercus, 3 –Picea; supplementary variables: see Fig 3; species codes–abbreviation of the species name consists of three letters of the genus name and three letters of the species name (see S2 Table). Only species observed more than once were included. Tree and shrub species of the T and S layers were excluded from the species list and their cover was included into the analysis as supplementary variables CovT1, CovT2, CovS1, CovS2.

Because of the lack of a tree canopy, the initiation stands were distinct from the other classes based on the presence of terrestrial light-demanding species: vascular plants, bryophytes and lichens (Fig 4A and 4B). These species accounted for the highest number of diagnostic and constant species in the initiation stands (Table 2). Species on CWD were more abundant in initiation stands (mostly lichens) and young stands (mostly bryophytes), where the amounts of substrate were higher when compared to the other stand age classes (Fig 4C, Table 1). In the young stands, epiphytic species were observed for the first time, and they occurred mostly on pine trunks (Fig 4D). Diagnostic and constant species of young stands were also species inhabiting CWD and pine trunks. Middle-aged stands had only two diagnostic species, which were epiphytes and constant species, which were associated with different substrates (Table 2). In pre-mature and mature stands, epiphytic species were observed on oak trunks (Fig 4D). *Quercus petraea* in the S1 layer also had the highest fidelity value in the mature stands, and the oldest stands also had the lowest number of constant species from pine epiphytes.

### Forest species and ancient forest species

Among the forest species the most abundant ones in all the age classes were species preferring forests and open land, i.e. group 2.1, according to the classification of Schmidt et al. [49], 53 species, 46% of the species pool (S1 and S2 Tables). Their cover was highest in all age classes in comparison to other forest species groups. In addition to their high distribution along a stand age gradient, they were most abundant as diagnostic species in initiation stands (Table 2). Moreover, in the initiation stands, among the diagnostic species, there were also species occurring in forests, but preferring open land (2.2) (S1 Table).

**Fig 5:**
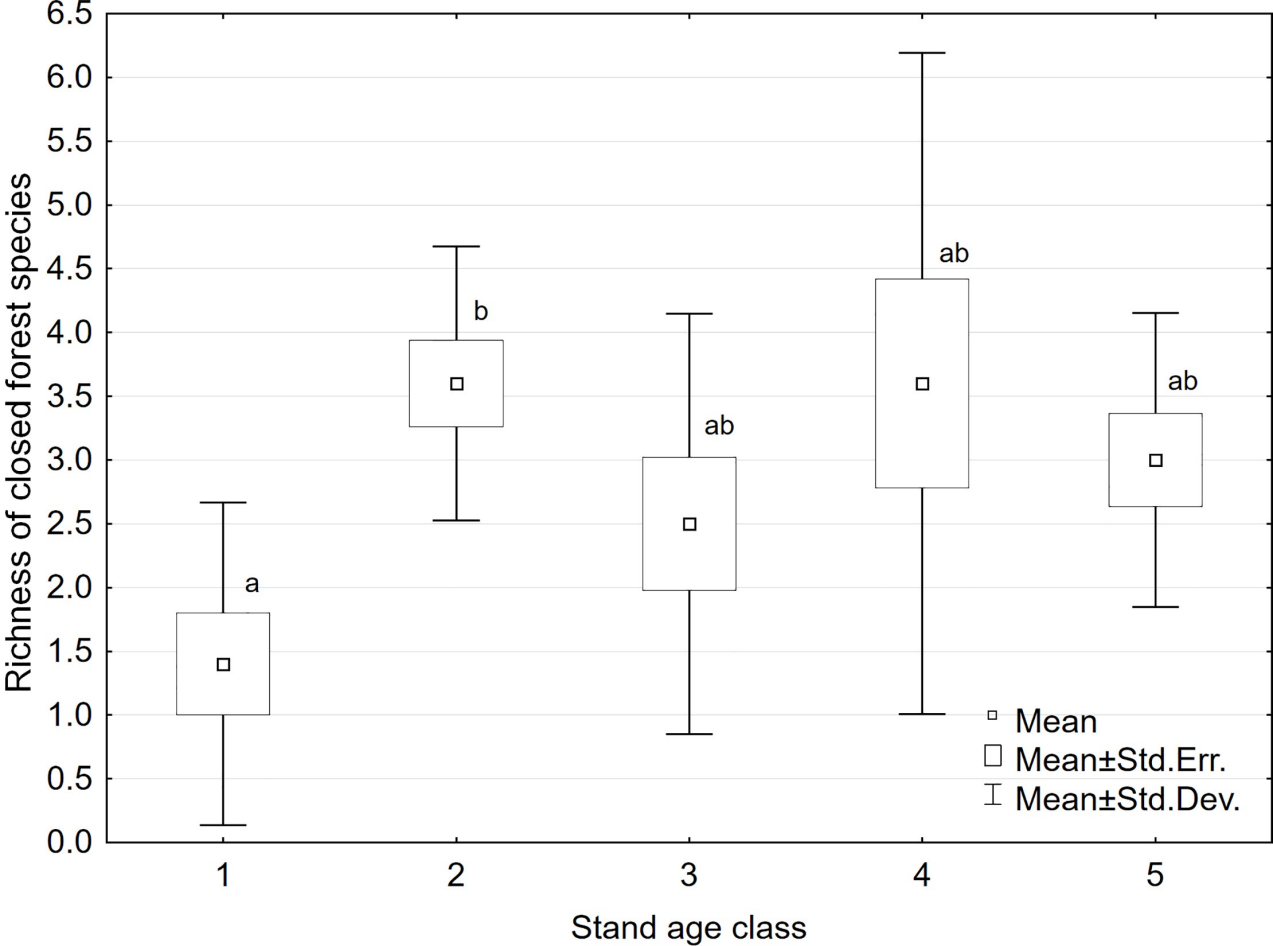
Richness of species restricted to closed forests (1.1). Means followed by the same letter are not significantly different according the Kruskal-Wallis test and multiple comparisons of average ranks as post-hoc test (p = 0.05).

Species restricted to closed forests (1.1) were observed in each age class. There were 21 species in the species pool (18%) (eight vascular plant species, eight bryophyte species, and five lichen species). Their richness was lowest in the initiation stands (Fig 5), increased in the young stands, and then remained relatively stable, with no correlation with stand age observed (S1 Table). This general increase of 1.1 species richness in young stands was especially caused by the increase of species inhabiting CWD and tree trunks (S1 Table). Among the closed forest species *Herzogiella seligeri* on CWD, and *Coenogonium pineti* and *Lophocolea heterophylla* on pine trunks were diagnostic species in young stands. *Chaenotheca ferruginea* on pine trunks and *Lophocolea heterophylla* on oak trunks were diagnostic species in mature stands (Table 2). In classes 2–5, *Lophocolea heterophylla* on CWD was a constant species.

**Fig 6:**
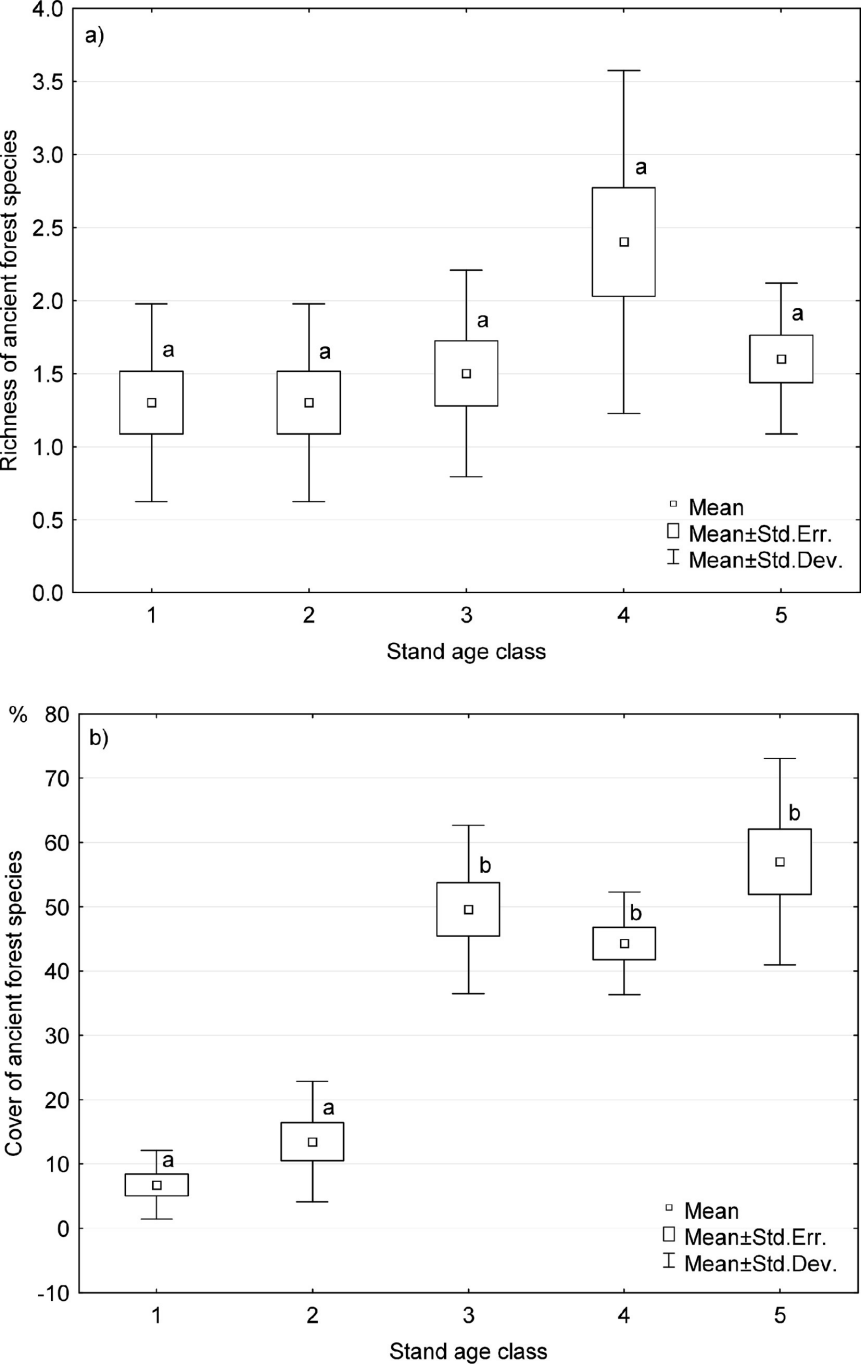
**Richness (a) and cover (b) of ancient forest species (AFS) in stand age classes.** Means followed by the same letter are not significantly different according the Kruskal-Wallis test and multiple comparisons of average ranks as post-hoc test (p = 0.05).

**Fig 7:**
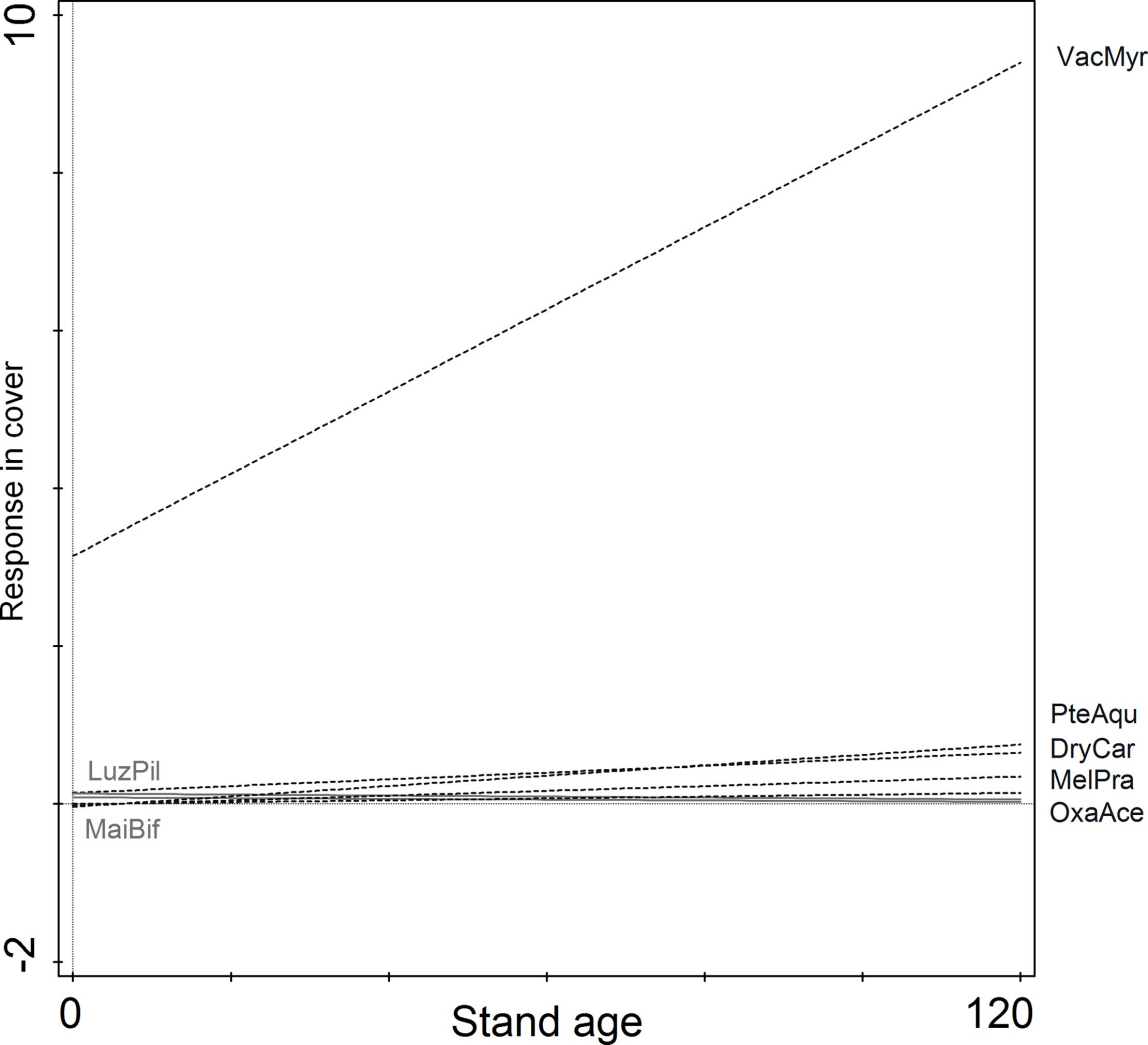
Quantitative changes of ancient forest species along time gradient. Model of species response was generated with the linear degree and Gaussian distribution as analysis options. Black dashed line–species of increasing cover; grey solid line–species of decreasing cover; species codes–abbreviation of the species name consists of three letters of the genus name and three letters of the species name (see S2 Table).

Seven ancient forest species (AFS) were found in all the studied plots. Their species pool in the successive age classes were as follows: 4, 4, 5, 7, and 4 (S2 Table). Their richness was low both in the studied area (2 species) as well as in specific age classes (1–2 species) (Fig 6A, S1 Table). The only AFS recorded in each plot was *V*. *myrtillus*. The frequency of the other AFS was low, and their distribution in age classes was rather random (S2 Table); however, *Dryopteris carthusiana* was the only diagnostic species in pre-mature stands (Table 2). No tendency of the AFS to co-occur was observed. As a group (at least four species among seven) AFS was found in only one plot of the pre-mature stands. Cover of AFS significantly increased with stand age (Fig 6B); however, the growing trend was largely associated with the increase in *V*. *myrtillus* cover (Fig 7).

## Discussion

### Dynamics of species diversity in Scots pine age-class forests

Although we sampled species that inhabited all the available substrates (soil, CWD, tree trunks), species pool and richness were relatively low–overall 116 species, average 25 per plot (S2 Table). This could be associated with the type of the studied habitats, which developed on acid soils [61]. Acid and nutrient-poor soils support only limited species pool which can adapt to such conditions, compared to the larger species pool on neutral or base rich soils [31,62]. In addition, Scots pine trunks are not the optimal host substrates for epiphytic species [63,64].

In managed forests, human-induced disturbances, such as large scale clear-cut logging, uniform vegetation structures over large areas and eliminate specialist species [9,35,65,66]. On the other hand, in the ground bryophyte layer of coniferous forests, a limited number of species is often observed, although with high cover [25,67]. However, relatively extended periods of forest habitat continuity facilitate the development of unique microclimate and vegetation structures which are beneficial to forest specialists [28]. In general, the species richness of cryptogams should be positively correlated with stand age [18,68]. In the examined forests, cryptogam species represented 61%–71% of the total species pool, depending on stand age class, but moss layer was predominated by a few species, especially *Pleurozium schreberi*. The occurrence of species on any other substrate, i.e. epiphytic and epixylous species, increases the level of biodiversity. In managed forests, the richness of epiphytic species is limited due to the removal of old trees through clear-cutting. During the life span of a tree stand (ca. 110 years in managed forests), some epiphytes are able to colonize the stand, but the occurrence of diaspore sources in the vicinity is a crucial precondition. In the case of epiphytes, old trees, which have rich biota, are such diaspore sources [18,69]. If there are no such propagule sources, the epiphytic species have a low chance of surviving in managed forests, even with favourable microclimates and the presence of suitable habitats [63,70]. The occurrence of trees of different ages is also a precondition for the maintenance of epiphyte diversity. In addition, the trees should be exposed to different light conditions, which is greatly limited in managed forests. Variability in the tree species in a forest complex is also beneficial for epiphytes. Broadleaved trees, particularly those with alkaline barks, are more beneficial to epiphytic organisms [63,71,72]. Results of previous studies reveal that even low number or sparsely distributed broadleaved tree species in a low canopy layer increase biodiversity in coniferous forests [73]. Indeed, in the studied stands, in older age classes, the epiphytic species occurred on oak trunks (Fig 4D). High species richness of epixylous organisms is influenced by the availability of large logs and snags, species diversity of CWD (derived from oaks, spruces, etc.), and their stages of decay [74–79]. In managed forests there is a shortage of appropriate substrates [13,74,75]. However, any amount of CWD, even if limited, is still valuable to the biodiversity of a forest [80]. In the examined forests, the majority of CWD was observed in young stands (Table 1), representing wood that was left behind after the first pre-commercial thinning. However, the CWD was rather small-sized, uniform and rapidly decomposing. Therefore, in older age classes, pre-mature and mature stands, the amount of CWD was minor. Consequently, the richness of the epixylous species was relatively low and short-lived (S1 Table). In the studied stands, the old trees available for specialist species and also the amount of the decaying substrates did not increase with increase in stand age. Even if a specific forest microclimate develops, there are no suitable substrates for specialists, such as epiphytes and epixylous species, to colonize. The decreasing potential for long-term colonization of tree trunks and CWD by such species results in vegetation being mainly composed of common species able to grow on diverse substrates (Fig 2). Numerous cryptogam species are not selective with regard to substrates [66], and such a trait facilitates their survival in the changing environment of managed forests. However, biodiversity in forests which do not host specialists remains low [65,81].

The disturbance caused by the tree rotation in managed stands limits the colonization and establishment of epiphytes and epixylous species, but facilitates the establishment of light-demanding species on bare soil in initiation stands (Fig 4). The removal of mature trees is followed by mechanical soil preparation (ploughing), which disrupts the forest floor cover considerably. The disruption of compact vegetation cover and upper soil layers is an opportunity for colonizers to establish their populations [21,41,82]. Initiation stands planted after clear-cutting are colonized by non-forest species, which take advantage of suitable light conditions and limited competition [20,22,24,25]. The presence of non-forest species increases the overall species pool in a forest complex. However, in close-canopy stands, light-demanding species do not find suitable habitat conditions and retreat [25,67].

Diversity patterns vary depending on the type of forest ecosystem, their management, and disturbances [83]. In managed forests a frequently described pattern of diversity changes is an increase in the species pool and richness after clear-cut logging [19,20,21,35]. Such a pattern was revealed only partly in the present study. The species pool of initiation and young stands was higher, but the species richness was similar in age classes. Increase of species richness after a stand removal was revealed when terrestrial species were analyzed [19,20,21,35]. In the studied forests, the richness of terrestrial species (mainly lichens and vascular plants) also increased in initiation stands. However, the species richness of subsequent age classes was not significantly different. This pattern resulted from taking into account species of different substrates. Terrestrial colonizers, epixylous and epiphytic species were not equally distributed in age classes. They exchanged during the stand growth, which stabilized the level of species richness. After the removal of old trees, the richness of terrestrial, non-forest species increased, but at the same time epiphytes disappeared. After canopy formation, light-demanding species declined, but epiphytes re-appeared and epixylous species increased on CWD. With the growth and maturation of the stand, the species richness did not increase, due to the minor participation of forest specialists.

### Forest species in managed ancient forests

Forest management activities promote non-forest species. In forests managed by clear-cutting, species tolerant to both forest and non-forest conditions have the greatest chance of surviving, and they are major components of the plant community [84]. Scots pine forests are dominated by species that are not “true” forest species [25]. In our study we observed that species occurring both in forests and open land, according to the Schmidt et al. [49] classification (group 2.1), represented a considerable proportion of species richness and cover (S1 Table). In the case of closed forest species (group 1.1), there were 21 species in the study area, which represented 18% of the species pool. Their richness in the initiation stands was low, and increased slightly after canopy closure. However, growing stand age was not correlated with further increase in closed forest species richness (Fig 5, S1 Table). The colonization of young stands by closed forest species could indicate the role of CWD, which was most abundant in young stands. In the studied pre-mature and mature stands, where the amount of CWD was low, the richness of forest specialists was also low.

Ancient forest species *sensu* Hermy et al. [17] were rather rare in the studied forest with long habitat continuity. Only seven ancient forest species were observed, 1–2 species per plot (S2 Table). Ancient forest species, which may occur in Scots pine forests, represent only a small part of the ancient forest species pool, because AFS lists were prepared based on rather fertile broadleaved forest studies [17,73,85,86]. However, low number of forest specialists and participation of species tolerant to non-forest conditions may also be a general feature of the acidophilous pine forest [87]. Analysis of the occurrence of ancient forest species in all Polish forests revealed that in *Dicrano-Pinion* communities only four AFS were common and had a tendency to co-occur: *Vaccinium myrtillus*, *Pteridium aquilinum*, *Melampyrum pretense*, and *Luzula pilosa* [85]. At the same time, *V*. *myrtillus* was common in both ancient and recent forests and only considerably more abundant in ancient forests [87]. In northwest Germany most of the acidophilous species recorded in forest communities were indifferent in terms of forest continuity [64,86]. Moreover, direct comparisons of ancient and secondary Scots pine forests revealed that species typical of open habitats, such as *Calluna vulgaris* and *Pohlia nutans*, were also indicators of ancient forests [31]. Whereas, closed forest species considered typical of Scots pine forests, i.e. species of the family *Pyrolaceae* Lindl. (actually included in *Ericaceae* Juss.), were most often observed in recent forests [15]. Actually, Scots pine dominated forests, even with high number of plant species, are not suitable for species preferring closed forest habitats [73]. This may be associated with the high transparency of Scots pine crowns. Pine crowns enable high light transmittance to the understory [88], which decreases ground and air moisture levels.

According to previous studies, managed forests, even mature stands before logging, may still be too young for specialist species [11,41]. However, spontaneous succession on oligotrophic sites with Scots pine forest as a terminal stage lasts about 140 years [89]. The oldest studied forests were of about 100 years, so there was enough time for rebuilding forest species composition. Moreover, in managed forests the time required for the development of the forest microclimate is shorter, because some natural long-lasting processes are modified or replaced by human interference, i.e. canopy formation and tree stem exclusion [19]. Therefore, the low richness and lack of species specific for the oldest stands may result from the lack of species sources (not managed old forests) in the vicinity of maturing stands [30].

## Conclusions

In the studied Scots pine ancient forests, species pool and richness were relatively low, and the vegetation consisted mostly of generalist species, which tolerate both forest and non-forest conditions. Age class forestry, based on clear-cutting, does not favour epiphytes and epixylous species. However, stand removal is beneficial to terrestrial light-demanding species. Each stand age class provided somewhat different environmental niches. Initiation stands were optimal places for non-forest species, which vanished after tree canopy closure. Young stands were preferred by species that exploited CWD niches, because in young stands coarse woody debris was the most abundant substrate. In closed-canopy stands (classes 2–5), epiphytes on tree trunks occurred. Bryophytes and lichens of closed forests (1.1) inhabited different substrates, and they were not associated exclusively with the oldest stands. In general, contrary to expectations, the oldest stands had only a few diagnostic species and were not rich in forest specialists. The low number of forest specialists in the oldest stands may be a general feature of acidophilus pine forests or a result of the lack of species sources in the vicinity of maturing pine stands. A frequently described pattern of diversity changes in managed forests is an increase in the species pool and species richness after clear-cut logging. In the present study we obtained a higher species pool in initiation and young stands, but species richness in subsequent age classes was not significantly different. This resulted from taking into account species of different substrates. These species were not equally distributed in age classes. They exchanged during the stand growth, which stabilized the level of species richness.

## Supporting information

S1 Table Mean values of variables analyzed in stand age classes and the Spearman coefficient (R) between the variables and tree stand age. Stand age classes: 1 –initiation stands (4–10 years), 2 –young stands (20–35 years), 3 –middle-aged stands (45–60 years), 4 –pre-mature stands (70–85 years), 5 –mature stands (95–110 years); *H*, *p*–value of statistics for the Kruskal-Wallis test; R–Spearman's rank correlation coefficient calculated with all stand age classes (bold means statistical significance); T1 –high tree layer, T2 –low tree layer, S1 –high shrub layer, S2 –low shrub layer, H–herb layer, M–bryophyte-lichen layer; species category: 1.1 –species restricted to closed forests, 1.2 –species preferring forest edges and clearings, 2.1 –species occurring in forests and in open land, 2.2 –species occurring in forests, but preferring open land, AFS–ancient forest species; LEIV–light index, FEIV–moisture index, REIV–reaction index, NEIV–nitrogen index; indices based on ecological indicator values for species; means followed by the same letter are not significantly different according the Kruskal-Wallis test and multiple comparisons of average ranks as post-hoc test (p = 0.05).
(DOCX)

S2 Table Percentage frequency of all species recorded in stand age classes. Stand age classes: 1 –initiation stands (4–10 years), 2 –young stands (20–35 years), 3 –middle-aged stands (45–60 years), 4 –pre-mature stands (70–85 years), 5 –mature stands (95–110 years); layers/substrates: T1 –high tree layer, T2 –low tree layer, S1 –high shrub layer, S2 –low shrub layer, H–herb layer, M–bryophyte-lichen layer, CWD–coarse woody debris, Pinus, Quercus, Picea–trunks as substrates for epiphytes; species category: 1.1 –species restricted to closed forests, 1.2 –species preferring forest edges and clearings, 2.1 –species occurring in forests and in open land, 2.2 –species occurring in forests, but preferring open land, AFS–ancient forest species.
(DOCX)

S1 Database Full data recorded for each sampling square in the fieldwork. (XLSX)

## References

[1] Matías L, Jump AS. Interactions between growth, demography and biotic interactions in determining species range limits in a warming world: the case of *Pinus sylvestris*. For Ecol Manage. 2012; 282: 10–22. 10.1016/j.foreco.2012.06.053

[2] Houston Durrant T, de Rigo D, Caudullo G. *Pinus sylvestris* in Europe: distribution, habitat, usage and threats In: San-Miguel-Ayanz J, de Rigo D, Caudullo G, Houston Durrant T, Mauri A, editors. European Atlas of Forest Tree Species. Luxembourg: Publ Off EU 2016; pp. e016b94+

[3] Mason WL, Alía R. Current and future status of Scots pine (*Pinus sylvestris* L.) forests in Europe. For Syst. 2000; 9(S1): 317–335.

[4] Spiecker H. Silvicultural management in maintaining biodiversity and resistance of forests in Europe–temperate zone. J Environ Manage. 2003; 67(1): 55–65. 10.1016/S0301-4797(02)00188-3 12659804

[5] Leuschner C, Ellenberg H. Ecology of Central European forests: vegetation ecology of Central Europe, vol 1 Berlin: Springer; 2017.

[6] Kelly DL, Connolly A. A review of the plant communities associated with Scots pine (*Pinus sylvestris* L.) in Europe, and an evaluation of putative indicator/specialist species. For Syst. 2000; 9(S1): 15–39.

[7] Matuszkiewicz JM. Zespoły leśne Polski. Warszawa: Wydawnictwo Naukowe PWN; 2001.

[8] Kącki Z, Czarniecka M, Swacha G. Statistical determination of diagnostic, constant and dominant species of the higher vegetation units of Poland. Monogr Bot. 2013; 103: 1–267. 10.5586/mb.2013.001

[9] Christensen M, Emborg J. Biodiversity in natural versus managed forest in Denmark. For Ecol Manage. 1996; 85(1–3): 47–51. 10.1016/S0378-1127(96)03749-8

[10] Marcos JA, Marcos E, Taboada A, Tárrega R. Comparison of community structure and soil characteristics in different aged Pinus sylvestris plantations and a natural pine forest. For Ecol Manage. 2007; 247(1–3): 35–42. 10.1016/j.foreco.2007.04.022

[11] Paillet Y, Bergès L, Hjältén J, Ódor P, Avon C, Bernhardt‐Römermann M, et al Biodiversity differences between managed and unmanaged forests: meta‐analysis of species richness in Europe. Conserv Biol. 2010; 24(1): 101–112. 10.1111/j.1523-1739.2009.01399.x 20121845

[12] Chaudhary A, Burivalova Z, Koh LP, Hellweg S. Impact of forest management on species richness: global meta-analysis and economic trade-offs. Sci Rep. 2016; 6: 23954 10.1038/srep23954 27040604 PMC4819217

[13] del Río M, Bravo-Oviedo JAB, Pretzsch H, Löf M, Ruiz-Peinado R. A review of thinning effects on Scots pine stands: From growth and yield to new challenges under global change. For Syst. 2017; 26(2). 10.5424/fs/2017262-11325

[14] Janssen P, Bec S, Fuhr M, Taberlet P, Brun JJ, Bouget C. Present conditions may mediate the legacy effect of past land‐use changes on species richness and composition of above‐and below‐ground assemblages. J Ecol. 2018; 106(1): 306–318. 10.1111/1365-2745.12808

[15] Matuszkiewicz JM, Kowalska A, Kozłowska A, Roo-Zielińska E, Solon J. Differences in plant-species composition, richness and community structure in ancient and post-agricultural pine forests in central Poland. For Ecol Manage. 2013; 310: 567–576. 10.1016/j.foreco.2013.08.060

[16] Hermy M, Verheyen K. Legacies of the past in the present-day forest biodiversity: a review of past land-use effects on forest plant species composition and diversity In: Nakashizuka T, editor. Sustainability and Diversity of Forest Ecosystems, Ecological Research. Japan: Springer; 2007 pp. 361–371. 10.1007/978-4-431-73238-9_1.

[17] Hermy M, Honnay O, Firbank L, Grashof-Bokdam C, Lawesson JE. An ecological comparison between ancient and other forest plant species of Europe, and the implications for forest conservation. Biol Conserv. 1999; 91(1): 9–22. 10.1016/S0006-3207(99)00045-2

[18] Hofmeister J, Hošek J, Brabec M, Dvořák D, Beran M, Deckerová H, et al Value of old forest attributes related to cryptogam species richness in temperate forests: A quantitative assessment. Ecol Indic. 2015; 57: 497–504. 10.1016/j.ecolind.2015.05.015

[19] Stefańska-Krzaczek E. Species diversity across the successional gradient of managed Scots pine stands in oligotrophic sites (SW Poland). J For Sci. 2012; 58(8): 345–356.

[20] Stefańska-Krzaczek E, Szymura TH. Species diversity of forest floor vegetation in age gradient of managed scots pine stands. Balt For. 2015; 21(2): 233–243.

[21] Pykälä J. Immediate increase in plant species richness after clear‐cutting of boreal herb‐rich forests. Appl Veg Sci. 2004; 7(1): 29–34. 10.1111/j.1654-109X.2004.tb00592.x

[22] Marozas V, Grigaitis V, Brazaitis G. Edge effect on ground vegetation in clear-cut edges of pine-dominated forests. Scand J Forest Res. 2005; 20(S6): 43–48. 10.1080/14004080510040986

[23] Widenfalk O, Weslien J. Plant species richness in managed boreal forests–Effects of stand succession and thinning. For Ecol Manage. 2009; 257(5): 1386–1394. 10.1016/j.foreco.2008.12.010

[24] Stefańska-Krzaczek E, Fałtynowicz W. Increase of *Cladonia* species diversity as a consequence of clear-cutting in nutrient-poor forest sites. Sylwan. 2013; 157(12): 929–936.

[25] Stefańska-Krzaczek E, Staniaszek-Kik M, Fałtynowicz W. Positive aspects of clear-cut logging? Ground bryophyte diversity along the age gradient of managed *Pinus sylvestris* stands. Cryptogam Bryol. 2016; 37(2):181–198. 10.7872/cryb/v37.iss2.2016.181

[26] Nordén B, Appelqvist T. Conceptual problems of ecological continuity and its bioindicators. Biodivers Conserv. 2001; 10(5): 779–791. 10.1023/A:1016675103935

[27] Kolb A, Diekmann M. Effects of environment, habitat configuration and forest continuity on the distribution of forest plant species. J Veg Sci. 2004; 15(2): 199–208. 10.1111/j.1654-1103.2004.tb02255.x

[28] Nordén B, Dahlberg A, Brandrud TE, Fritz Ö, Ejrnaes R, Ovaskainen O. Effects of ecological continuity on species richness and composition in forests and woodlands: a review. Ecoscience. 2014; 21(1): 34–45. 10.2980/21-1-3667

[29] Summers RW, Mavor RA, MacLennan AM, Rebecca GW. The structure of ancient native pinewoods and other woodlands in the Highlands of Scotland. For Ecol Manage. 1999; 119(1–3): 231–245. 10.1016/S0378-1127(98)00515-5

[30] Orczewska A, Fernes M. Migration of herb layer species into the poorest post-agricultural pine woods adjacent to ancient pine forests. Pol J Ecol 2011; 59(1): 75–85.

[31] Kowalska A, Matuszkiewicz JM, Solon J, Kozłowska A. Indicators of ancient forests in nutrient-deficient pine habitats. Silva Fenn. 2017; 51(1). 10.14214/sf.1684

[32] Tonteri T, Salemaa M, Rautio P, Hallikainen V, Korpela L, Merilä P. Forest management regulates temporal change in the cover of boreal plant species. For Ecol Manage. 2016; 381: 115–124. 10.1016/j.foreco.2016.09.015

[33] Puchniarski TH. Sosna zwyczajna: hodowla i ochrona. Warszawa: PWRiL; 2008.

[34] Bråkenhielm S, Liu Q. Long-term effects of clear-felling on vegetation dynamics and species diversity in a boreal pine forest. Biodivers Conserv 1998; 7(2): 207–220. 10.1023/A:1008836502640

[35] Ujházy K, Hederová L, Máliš F, Ujházyová M, Bosela M, Čiliak M. Overstorey dynamics controls plant diversity in age-class temperate forests. For Ecol Manage. 2017; 391: 96–105. 10.1016/j.foreco.2017.02.010

[36] Česonienė L, Daubaras R, Kaškonas P, Kaškonienė V, Maruška AS, Tiso N, et al Initial impact of clear-cut logging on dynamics of understory vascular plants and pollinators in Scots pine-dominated forests in Lithuania. Turk J Agric For. 2018; 42: 1–12. 10.3906/tar-1804-71

[37] Aussenac G. Interactions between forest stands and microclimate: ecophysiological aspects and consequences for silviculture. Ann For Sci. 2000; 57(3): 287–301. 10.1051/forest:2000119

[38] Nelson CR, Halpern CB. Short-term effects of timber harvest and forest edges on ground-layer mosses and liverworts. Can J Bot. 2005; 83(6): 610–620. 10.1139/b05-036

[39] Thomas SC, Halpern CB, Falk DA, Liguori DA, Austin KA. Plant diversity in managed forests: understory responses to thinning and fertilization. Ecol Appl. 1999; 9(3): 864–879. 10.1890/1051-0761(1999)009[0864:PDIMFU]2.0.CO;2

[40] Ares A, Neill AR, Puettmann KJ. Understory abundance, species diversity and functional attribute response to thinning in coniferous stands. For Ecol Manage. 2010; 260(7): 1104–1113. 10.1016/j.foreco.2010.06.023

[41] Duguid MC, Ashton MS. A meta-analysis of the effect of forest management for timber on understory plant species diversity in temperate forests. For Ecol Manage. 2013; 303: 81–90. 10.1016/j.foreco.2013.04.009

[42] Bernes C, Jonsson BG, Junninen K, Lõhmus A, Macdonald E, Müller J, et al What is the impact of active management on biodiversity in boreal and temperate forests set aside for conservation or restoration? A systematic map. Environmental Evidence. 2015; 4(1): 25 10.1186/s13750-015-0050-7

[43] Augusto L, Dupouey JL, Ranger J. Effects of tree species on understory vegetation and environmental conditions in temperate forests. Ann For Sci. 2003; 60(8): 823–831. 10.1051/forest:2003077

[44] Barbier S, Gosselin F, Balandier P. Influence of tree species on understory vegetation diversity and mechanisms involved–a critical review for temperate and boreal forests. For Ecol Manage. 2008; 254(1): 1–15.

[45] Szymura TH, Dunajski A, Ruczakowska AM. Zmiany powierzchni lasów na obszarze Karkonoskiego Parku Narodowego w okresie 1747–1977. Opera Carconotica. 2010; 48, supl.1: 159–166.

[46] Szymura TH, Murak S, Szymura M, Raduła MW. Changes in forest cover in Sudety Mountains during the last 250 years: patterns, drivers, and landscape-scale implications for nature conservation. Acta Soc Bot Pol. 2018; 87(1): 1–14. 10.5586/asbp.3576

[47] IUSS Working Group WRB. World Reference Base for Soil Resources 2014, update 2015 International soil classification system for naming soils and creating legends for soil maps. Rome: FAO, World Soil Resources Reports 2015; 106.

[48] Ellenberg H, Weber H, Düll R, Wirth V, Werner W, Paulißen D. Zeigerwerte von Pflanzen in Mitteleuropa. Scr Geobot. 1992; 18: 5–258.

[49] Schmidt M, Kriebitzsch WU, Ewald J. (eds). Waldartenlisten der Farn-und Blütenpflanzen, Moose und Flechten Deutschlands. BfN-Skripten. 2011; 299.

[50] Hill MO, Preston CD, Bosanquet SD, Roy DB. BRYOATT: attributes of British and Irish mosses, liverworts and hornworts. Cambridge: Centre for Ecology and Hydrology, 2007.

[51] Statsoft Inc. Electronic Statistics Textbook. Tulsa: OK, StatSoft; 2013. Available from: http://www.statsoft.com/textbook/ Cited 09 January 2019.

[52] Šmilauer P, Lepš J. Multivariate analysis of ecological data using CANOCO 5. Cambridge: Cambridge University Press; 2014.

[53] Tichý L, Holt J, Nejezchlebová M. JUICE program for management, analysis and classification of ecological data. 2nd Edition of the Program Manual. 2nd part. Brno: Vegetation Science Group, Masaryk University Brno; 2010.

[54] Tichý L, Holt J. JUICE Program for management, analysis and classification of ecological data. Program Manual. Brno: Masaryk University, Vegetation Science Group; 2006.

[55] Euro+Med PlantBase—the information resource for Euro-Mediterranean plant diversity. Available from: http://ww2.bgbm.org/EuroPlusMed/ [Cited 09 January 2019].

[56] Ochyra R, Żarnowiec J, Bednarek-Ochyra H. Census catalogue of Polish mosses. Kraków: W. Szafer Instiute of Botany; 2003.

[57] Stebel A. The mosses of the Beskidy Zachodnie as a paradigm of biological and environmental changes in the flora of the Polish Western Carpathians. Katowice-Poznań: Śląski Uniwersytet Medyczny & Wydawnictwo Sorus; 2006.

[58] Klama H. Systematic catalogue of Polish liverwort and hornwort taxa In: Szweykowski J, editor. An annotated checklist of Polish liverworts and hornworts. Kraków: W. Szafer Institute of Botany; 2006 pp. 83–100.

[59] Index Fungorum. Available from: http://www.indexfungorum.org [Cited 09 January 2019].

[60] MycoBank Database. Available from: http://www.mycobank.org [Cited 09 January 2019].

[61] Zawadzki S. Gleboznawstwo. Warszawa: PWRiL; 1999.

[62] Cornwell WK, Grubb PJ. Regional and local patterns in plant species richness with respect to resource availability. Oikos. 2003; 100(3): 417–428. 10.1034/j.1600-0706.2003.11697.x

[63] Ódor P, Király I, Tinya F, Bortignon F, Nascimbene J. Patterns and drivers of species composition of epiphytic bryophytes and lichens in managed temperate forests. For Ecol Manage. 2013; 306: 256–265. 10.1016/j.foreco.2013.07.001

[64] Mölder A, Schmidt M, Engel F, Schönfelder E, Schulz F. Bryophytes as indicators of ancient woodlands in Schleswig-Holstein (Northern Germany). Ecol Indic. 2015; 54: 12–30. 10.1016/j.ecolind.2015.01.044.

[65] Vellak K, Paal J. Diversity of bryophyte vegetation in some forest types in Estonia: a comparison of old unmanaged and managed forests. Biodiv Conserv. 1999; 8(12): 1595–1620. 10.1023/A:1008927501623

[66] Ardelean IV, Keller C, Scheidegger C. Effects of management on lichen species richness, ecological traits and community structure in the Rodnei Mountains National Park (Romania). PLoSONe. 2015;10(12): e0145808 10.1371/journal.pone.0145808 26717517 PMC4696781

[67] Boudreault C, Paquette M, Fenton NJ, Pothier D, Bergeron Y. Changes in bryophytes assemblages along a chronosequence in eastern boreal forest of Quebec. Can J Forest Res. 2018; 48(999): 1–14. 10.1139/cjfr-2017-0352

[68] Boudreault C, Drapeau P, Bouchard M, St-Laurent MH, Imbeau L, Bergeron Y. Contrasting responses of epiphytic and terricolous lichens to variations in forest characteristics in northern boreal ecosystems. Can J Forest Res. 2015; 45(5): 595–606. 10.1139/cjfr-2013-0529

[69] Dittrich S, Hauck M, Schweigatz D, Dörfler I, Hühne R, Bade C, et al Separating forest continuity from tree age effects on plant diversity in the ground and epiphyte vegetation of a Central European mountain spruce forest. Flora Morphol Distrib Funct Ecol Plants. 2013; 208(4): 238–246. 10.1016/j.flora.2013.03.006

[70] Kaufmann S, Hauck M, Leuschner C. Comparing the plant diversity of paired beech primeval and production forests: Management reduces cryptogam, but not vascular plant species richness. For Ecol and Manage. 2017; 400: 58–67. 10.1016/j.foreco.2017.05.043

[71] Wolseley P, Sanderson N, Thüs H, Carpenter D, Eggleton P. Patterns and drivers of lichen species composition in a NW-European lowland deciduous woodland complex. Biodivers and Conserv. 2017; 26(2): 401–419. 10.1007/s10531-016-1250-3

[72] Mežaka A, Brūelis G, Piterāns A. Tree and stand-scale factors affecting richness and composition of epiphytic bryophytes and lichens in deciduous woodland key habitats. Biodivers Conserv 2012; 21: 3221–3241. 10.1007/s10531-012-0361-8

[73] Müller J, Boch S, Prati D, Socher SA, Pommer U, Hessenmöller D, et al Effects of forest management on bryophyte species richness in Central European forests. For Ecol Manage. 2019, 432, 850–859. 10.1016/j.foreco.2018.10.019

[74] Madžule L, Brūmelis G, Tjarve D. Structures determining bryophyte species richness in a managed forest landscape in boreo-nemoral Europe. Biodivers Conserv. 2012; 21(2): 437–450. 10.1007/s10531-011-0192-z

[75] Táborská M, Procházková J, Lengyel A, Vrška T, Hort L, Ódor P. Wood-inhabiting bryophyte communities are influenced by different management intensities in the past. Biodivers Conserv. 2017; 26(12): 2893–2909. 10.1007/s10531-017-1395-8

[76] Humphrey JW, Davey S, Peace AJ, Ferris R, Harding K. Lichens and bryophyte communities of planted and semi-natural forests in Britain: the influence of site type, stand structure and deadwood. Biol Conserv. 2002; 107(2): 165–180. 10.1016/S0006-3207(02)00057-5

[77] Kumar P, Chen HY, Thomas SC, Shahi C. Effects of coarse woody debris on plant and lichen species composition in boreal forests. J Veg Sci. 2017; 28(2): 389–400. 10.1111/jvs.12485

[78] Lelli C, Bruun HH, Chiarucci A, Donati D, Frascaroli F, Fritz Ö, et al Biodiversity response to forest structure and management: Comparing species richness, conservation relevant species and functional diversity as metrics in forest conservation. For Ecol Manage. 2019; 432: 707–717. 10.1016/j.foreco.2018.09.057

[79] Staniaszek-Kik M, Żarnowiec J. Diversity of mosses on stumps and logs in the Karkonosze Mts (Sudetes Mts, Central Europe). Herzogia. 2018; 31(1): 70–87. 10.13158/099.031.0104

[80] Kruys N, Jonsson BG. Fine woody debris is important for species richness on logs in managed boreal spruce forests of northern Sweden. Can J Forest Res. 1999; 29(8): 1295–1299. 10.1139/x99-106

[81] Andersson LI, Hytteborn H. Bryophytes and decaying wood–a comparison between managed and natural forest. Ecography. 1991; 14(2): 121–130. 10.1111/j.1600-0587.1991.tb00642.x

[82] Paquette M, Boudreault C, Fenton N, Pothier D, Bergeron Y. Bryophyte species assemblages in fire and clear-cut origin boreal forests. For Ecol Manage. 2016; 359: 99–108. 10.1016/j.foreco.2015.09.031

[83] Roberts MR, Gilliam FS. Patterns and mechanisms of plant diversity in forested ecosystems: implications for forest management. Ecol Appl. 1995; 5(4): 969–977. 10.2307/2269348

[84] Baisheva EZ, Shirokikh PS, Martynenko VB. Effect of clear-cutting on bryophytes in pine forests of the South Urals. Arctoa. 2015; 24(2): 547–555. 10.15298/arctoa.24.47

[85] Stefańska-Krzaczek E, Kącki Z, Szypuła B. Coexistence of ancient forest species as an indicator of high species richness. For Ecol Manage. 2016; 365: 12–21. 10.1016/j.foreco.2016.01.012

[86] Schmidt M, Mölder A, Schönfelder E, Engel F, Schmiedel I, Culmsee H. Determining ancient woodland indicator plants for practical use: a new, approach developed in northwest Germany. For Ecol Manage. 2014; 330: 228–239. 10.1016/j.foreco.2014.06.043

[87] Matuszkiewicz JM, Kowalska A, Solon J, Degórski M, Kozłowska A, Roo-Zielińska E, et al Long-term evolution models of post-agricultural forests. Warszawa: IGiPZ PAN; 2013.

[88] Coote L, French LJ, Moore KM, Mitchell FJG, Kelly DL. Can plantation forests support plant species and communities of semi-natural woodland? For Ecol Manage. 2012; 283: 86–95. 10.1016/j.foreco.2012.07.013

[89] Faliński JB, Cieśliński S, Czyżewska K. Dynamic-floristic atlas of Jelonka reserve and adjacent areas. Distribution of vascular plant species, bryophytes and lichens on the abandoned farmlands during secondary succession. Phytocoenosis 1993; 5, Supplementum Cartographiae Geobotanicae 3: 3–139.

