# Supplementary material for: Species diversity patterns in managed Scots pine stands in ancient forest sites
Source: PLoS One. 2019 Jul 11;14(7):e0219620. doi: 10.1371/journal.pone.0219620 (PMC6622550; doi:10.1371/journal.pone.0219620)
Supplement: S1 Table — Stand age classes: 1 –initiation stands (4–10 years), 2 –young stands (20–35 years), 3 –middle-aged stands (45–60 years), 4 –pre-mature stands (70–85 years), 5 –mature stands (95–110 years); H, p–value of statistics for the Kruskal-Wallis test; R–Spearman's rank correlation coefficient calculated with all stand age classes (bold means statistical significance); T1 –high tree layer, T2 –low tree layer, S1 –high shrub layer, S2 –low shrub layer, H–herb layer, M–bryophyte-lichen layer; species category: 1.1 –species restricted to closed forests, 1.2 –species preferring forest edges and clearings, 2.1 –species occurring in forests and in open land, 2.2 –species occurring in forests, but preferring open land, AFS–ancient forest species; LEIV–light index, FEIV–moisture index, REIV–reaction index, NEIV–nitrogen index; indices based on ecological indicator values for species; means followed by the same letter are not significantly different according the Kruskal-Wallis test and multiple comparisons of average ranks as post-hoc test (p = 0.05). (DOCX) [file pone.0219620.s001.docx]

**S1 Table. Mean values of variables analysed in stand age classes and Spearman coefficient (R) between variables and tree stand age.**

|  | Class 1 | Class 2 | Class 3 | Class 4 | Class 5 | *H* | *p* | R |
| --- | --- | --- | --- | --- | --- | --- | --- | --- |
| Species richness; T1 | 0.00 | 1.10a | 1.10a | 1.10a | 1.10a | 38.04 | 0.000 | **0.617** |
| Species cover; T1 | 0.00 | 70.53a | 60.51a | 60.14a | 58.51a | 31.02 | 0.000 | 0.255 |
| Species richness; T2 | 0.10ab | 0.00a | 0.30ab | 0.40ab | 1.00b | 15.51 | 0.004 | **0.552** |
| Species cover; T2 | 1.20a | 0.00a | 4.40a | 11.30a | 10.55a | 13.17 | 0.011 | **0.522** |
| Species richness; S1 | 0.00a | 0.40ab | 0.20a | 1.50b | 1.60b | 28.13 | 0.000 | **0.684** |
| Species cover; S1 | 0.00a | 3.37ab | 0.40ab | 9.07bc | 21.19c | 28.65 | 0.000 | **0.667** |
| Species richness; S2 | 2.90b | 1.20a | 2.10ab | 1.40a | 1.40a | 16.39 | 0.003 | **-0.386** |
| Species cover; S2 | 54.70 | 3.56a | 4.81a | 3.18a | 2.63a | 24.70 | 0.000 | **-0.489** |
| Species richness; H | 7.90a | 5.30b | 5.80ab | 6.70ab | 4.30b | 15.09 | 0.005 | **-0.356** |
| Species cover; H | 29.53ab | 22.95a | 52.66bc | 48.6bc9 | 57.59c | 27.24 | 0.000 | **0.636** |
| Species richness; M | 11.40a | 7.80ab | 6.10b | 5.60b | 8.40ab | 14.00 | 0.007 | **-0.282** |
| Species cover; M | 12.49 | 47.34a | 63.18a | 59.58a | 48.08a | 24.78 | 0.000 | **0.499** |
| Species richness; M, bryophytes | 8.40a | 6.90a | 6.00a | 5.40a | 7.80a | 6.42 | 0.170 | -0.111 |
| Species cover; M, bryophytes | 7.19 | 47.33a | 63.18a | 59.58a | 48.08a | 24.97 | 0.000 | **0.500** |
| Species richness; M, lichens | 3.00a | 0.90ab | 0.10b | 0.20b | 0.60b | 26.10 | 0.000 | **-0.531** |
| Species cover; M, lichens | 5.64 | 0.01a | 0.00a | 0.00a | 0.00a | 44.84 | 0.000 | **-0.700** |
| Species richness; CWD | 8.40ab | 12.70a | 6.80b | 5.70b | 7.40ab | 13.05 | 0.011 | -0.246 |
| Species richness; CWD, bryophytes | 3.40ab | 8.80c | 5.20abc | 4.30b | 4.90abc | 14.65 | 0.006 | -0.037 |
| Species richness; CWD, lichens | 5.00a | 3.90ab | 1.60bc | 1.40c | 2.50ac | 18.27 | 0.001 | **-0.450** |
| Species richness; Pinus trunks | 0.00a | 8.60b | 5.60b | 5.00b | 4.10ab | 25.89 | 0.000 | 0.213 |
| Species richness; Pinus trunks, bryophytes | 0.00a | 3.60b | 1.60ab | 1.20ab | 1.40ab | 23.46 | 0.000 | 0.076 |
| Species richness; Pinus trunks, lichens | 0.00a | 5.00b | 4.00b | 3.80b | 2.70ab | 23.40 | 0.000 | **0.281** |
| Species richness; Quercus trunks | 0.00a | 0.50a | 1.00a | 0.40a | 4.40a | 9.97 | 0.041 | **0.302** |
| Species richness; Quercus trunks, bryophytes | 0.00a | 0.30a | 0.70a | 0.30a | 3.20a | 10.06 | 0.040 | **0.303** |
| Species richness; Quercus trunks, lichens | 0.00a | 0.20a | 0.30a | 0.10a | 1.20a | 9.49 | 0.050 | **0.287** |
| Species richness; Picea trunks | 0.00a | 0.00a | 0.00a | 0.90a | 0.60a | 11.60 | 0.021 | **0.383** |
| Species richness; Picea trunks, bryophytes | 0.00a | 0.00a | 0.00a | 0.60a | 0.50a | 11.47 | 0.022 | **0.386** |
| Species richness; Picea trunks, lichens | 0.00a | 0.00a | 0.00a | 0.30a | 0.10a | 5.63 | 0.229 | 0.247 |
| Species richness; trunks | 0.00 | 8.80a | 6.30a | 5.90a | 8.90a | 26.48 | 0.000 | **0.451** |
| Species richness; trunks, bryophytes | 0.00a | 3.80b | 2.00ab | 1.80ab | 5.10b | 25.57 | 0.000 | **0.398** |
| Species richness; trunks, lichens | 0.00 | 5.00a | 4.30a | 4.10a | 3.80a | 23.88 | 0.000 | **0.392** |
| Species richness; tree and shrub species | 3.50a | 3.40a | 3.90a | 4.20a | 3.80a | 3.27 | 0.514 | 0.151 |
| Species richness; vascular non tree species | 6.00a | 3.60a | 3.90ab | 4.90ab | 3.20b | 12.79 | 0.012 | **-0.309** |
| Species richness; bryophyte species | 9.60abc | 12.90ac | 9.50abc | 8.00b | 12.80c | 17.60 | 0.002 | 0.074 |
| Species richness; lichen species | 6.80a | 7.20a | 5.80a | 5.10a | 5.50a | 4.62 | 0.328 | -0.245 |
| Species richness; vascular non tree species and terrestrial cryptogams | 17.40 | 11.40a | 10.00a | 10.50a | 11.60a | 16.85 | 0.002 | **-0.393** |
| Species richness; all | 25.90a | 27.10a | 23.10a | 22.20a | 25.30a | 5.78 | 0.216 | -0.180 |
| Species richness; AFS | 1.30a | 1.30a | 1.50a | 2.40a | 1.60a | 10.93 | 0.027 | **0.323** |
| Species cover; AFS | 6.76a | 13.47a | 49.57b | 44.30b | 57.00b | 36.96 | 0.000 | **0.803** |
| Species richness; 1.1, H | 0.40a | 0.30a | 0.50a | 1.50a | 0.50a | 4.55 | 0.337 | 0.197 |
| Species cover; 1.1, H | 0.05a | 1.01a | 0.74a | 1.33a | 2.12a | 4.04 | 0.401 | 0.184 |
| Species richness; 2.1, H | 4.90a | 3.20b | 3.30ab | 3.20ab | 2.60b | 17.96 | 0.001 | **-0.517** |
| Species cover; 2.1, H | 27.96ab | 21.98a | 51.99c | 47.54bc | 56.74c | 27.99 | 0.000 | **0.645** |
| Species richness; 2.2, H | 0.20a | 0.00a | 0.00a | 0.00a | 0.00a | 8.17 | 0.086 | **-0.319** |
| Species cover; 2.2, H | 0.01a | 0.00a | 0.00a | 0.00a | 0.00a | 4.00 | 0.406 | -0.243 |
| Species richness; 1.1, M | 0.20a | 0.70a | 0.20a | 0.40a | 0.70a | 4.88 | 0.300 | 0.139 |
| Species cover; 1.1, M | 0.04a | 0.08a | 0.01a | 0.00a | 0.13a | 6.16 | 0.188 | 0.016 |
| Species richness; 1.2, M | 0.10a | 0.10a | 0.00a | 0.00a | 0.00a | 3.06 | 0.547 | -0.220 |
| Species cover; 1.2, M | 0.00a | 0.00a | 0.00a | 0.00a | 0.00a | 0.00 | 1.000 |  |
| Species richness; 2.1, M | 8.30a | 6.60a | 5.90a | 5.10a | 7.60a | 7.92 | 0.095 | -0.138 |
| Species cover; 2.1, M | 7.60 | 47.28a | 63.18a | 59.56a | 48.00a | 25.17 | 0.000 | **0.498** |
| Species richness; 2.2, M | 2.60 | 0.10a | 0.00a | 0.10a | 0.00a | 41.53 | 0.000 | **-0.661** |
| Species cover; 2.2, M | 5.26 | 0.00a | 0.00a | 0.03a | 0.00a | 38.74 | 0.000 | **-0.600** |
| Species richness; 1.1, CWD | 0.80a | 2.50b | 1.30ab | 1.30ab | 1.20ab | 9.92 | 0.042 | -0.003 |
| Species richness; 1.2, CWD | 0.30a | 0.20a | 0.00a | 0.00a | 0.00a | 6.39 | 0.172 | **-0.359** |
| Species richness; 2.1, CWD | 6.00ab | 9.10a | 4.90ab | 4.10b | 5.70ab | 11.13 | 0.025 | -0.205 |
| Species richness; 2.2, CWD | 0.90a | 0.40a | 0.10a | 0.00a | 0.00a | 16.38 | 0.003 | **-0.510** |
| Species richness; 1.1, trunks | 0.00a | 2.20b | 1.30ab | 1.10ab | 1.70b | 22.39 | 0.000 | 0.258 |
| Species richness; 1.2, trunks | 0.00a | 0.00a | 0.00a | 0.00a | 0.10a | 4.00 | 0.406 | 0.154 |
| Species richness; 2.1, trunks | 0.00a | 5.20b | 3.00ab | 3.80b | 5.70b | 27.35 | 0.000 | **0.529** |
| Species richness; 2.2, trunks | 0.00a | 0.30a | 0.60a | 0.10a | 0.10a | 13.02 | 0.011 | -0.003 |
| Species richness; 1.1, all | 1.40a | 3.60b | 2.50ab | 3.60ab | 3.00ab | 12.34 | 0.015 | 0.230 |
| Species richness; 1.2 all | 0.40a | 0.20a | 0.00a | 0.00a | 0.10a | 6.45 | 0.168 | **-0.324** |
| Species richness; 2.1 all | 16.00ab | 17.50a | 14.20ab | 13.00b | 16.60ab | 12.94 | 0.012 | -0.121 |
| Species richness; 2.2 all | 3.50 | 0.70a | 0.70a | 0.20a | 0.10a | 31.87 | 0.000 | **-0.719** |
| LEIV | 6.14 | 5.49a | 5.40a | 5.35a | 5.35a | 23.07 | 0.000 | **-0.525** |
| FEIV | 4.26a | 4.48a | 4.48a | 4.55a | 4.46a | 3.97 | 0.411 | 0.122 |
| REIV | 2.43a | 2.87b | 2.87b | 2.86b | 2.79ab | 15.38 | 0.004 | **0.337** |
| NEIV | 2.82a | 2.88a | 2.90a | 3.03a | 2.82a | 0.69 | 0.952 | -0.022 |

Stand age classes: 1 – initiation stands (4–10 years), 2 – young stands (20–35 years), 3 – middle-aged stands (45–60 years), 4 – pre-mature stands (70–85 years), 5 – mature stands (95–110 years); *H, p* – value of statistics for the Kruskal-Wallis test; R – Spearman's rank correlation coefficient calculated with all stand age classes (bold means statistical significance); T1 – high tree layer, T2 – low tree layer, S1 – high shrub layer, S2 – low shrub layer, H – herb layer, M – bryophyte-lichen layer; species category: 1.1 – species restricted to closed forests, 1.2 – species preferring forest edges and clearings, 2.1 – species occurring in forests and in open land, 2.2 – species occurring in forests, but preferring open land, AFS – ancient forest species; LEIV – light index, FEIV – moisture index, REIV – reaction index, NEIV – nitrogen index; indices based on ecological indicator values for species; means followed by the same letter are not significantly different according the Kruskal-Wallis test and multiple comparisons of average ranks as post-hoc test (p = 0.05).
