# Supplementary material for: Species diversity patterns in managed Scots pine stands in ancient forest sites
Source: PLoS One. 2019 Jul 11;14(7):e0219620. doi: 10.1371/journal.pone.0219620 (PMC6622550; doi:10.1371/journal.pone.0219620)
Supplement: S2 Table — Stand age classes: 1 –initiation stands (4–10 years), 2 –young stands (20–35 years), 3 –middle-aged stands (45–60 years), 4 –pre-mature stands (70–85 years), 5 –mature stands (95–110 years); layers/substrates: T1 –high tree layer, T2 –low tree layer, S1 –high shrub layer, S2 –low shrub layer, H–herb layer, M–bryophyte-lichen layer, CWD–coarse woody debris, Pinus, Quercus, Picea–trunks as substrates for epiphytes; species category: 1.1 –species restricted to closed forests, 1.2 –species preferring forest edges and clearings, 2.1 –species occurring in forests and in open land, 2.2 –species occurring in forests, but preferring open land, AFS–ancient forest species. (DOCX) [file pone.0219620.s002.docx]

**S2 Table. Percentage frequency of all species recorded in stand age classes**.

| Species | Layer/Substrate | Species category | Class 1 F% | Class 2 F% | Class 3 F% | Class 4 F% | Class 5 F% |
| --- | --- | --- | --- | --- | --- | --- | --- |
| *Betula pendula* | T1 |  |  |  |  |  | 10 |
| *Betula pubescens* | T1 |  |  | 10 |  |  |  |
| *Larix decidua* | T1 |  |  |  | 10 |  |  |
| *Pinus sylvestris* | T1 |  |  | 100 | 100 | 100 | 100 |
| *Quercus petraea* | T1 |  |  |  |  | 10 |  |
| *Fagus sylvatica* | T2 |  |  |  | 10 | 20 | 10 |
| *Picea abies* | T2 |  |  |  |  |  | 20 |
| *Quercus petraea* | T2 |  | 10 |  | 20 | 20 | 50 |
| *Quercus robur* | T2 |  |  |  |  |  | 20 |
| *Betula pendula* | S1 |  |  |  |  |  | 10 |
| *Fagus sylvatica* | S1 |  |  | 30 |  | 50 | 10 |
| *Picea abies* | S1 |  |  |  |  | 80 | 40 |
| *Quercus petraea* | S1 |  |  | 10 | 20 | 10 | 80 |
| *Quercus robur* | S1 |  |  |  |  |  | 10 |
| *Sorbus aucuparia* | S1 |  |  |  |  | 10 | 10 |
| *Betula pendula* | S2 |  | 90 |  | 30 | 10 | 10 |
| *Betula pubescens* | S2 |  |  | 10 | 10 |  |  |
| *Carpinus betulus* | S2 |  |  |  |  | 10 | 10 |
| *Fagus sylvatica* | S2 |  |  | 10 | 10 | 20 |  |
| *Frangula alnus* | S2 |  | 30 | 10 | 10 | 10 | 20 |
| *Larix decidua* | S2 |  |  |  | 10 |  |  |
| *Picea abies* | S2 |  |  | 60 | 20 | 30 | 20 |
| *Pinus sylvestris* | S2 |  | 100 |  | 10 |  |  |
| *Populus tremula* | S2 |  | 10 |  |  |  |  |
| *Prunus serotina* | S2 |  | 10 | 10 |  |  |  |
| *Quercus petraea* | S2 |  | 30 | 20 | 40 | 30 | 70 |
| *Quercus robur* | S2 |  |  |  | 20 | 10 | 10 |
| *Sorbus aucuparia* | S2 |  | 20 |  | 50 | 20 |  |
| *Abies alba* | H |  |  |  |  | 10 |  |
| *Avenella flexuosa* | H | 2.1 | 100 | 100 | 100 | 90 | 70 |
| *Betula pendula* | H |  | 70 | 10 | 20 | 10 |  |
| *Betula pubescens* | H |  |  | 10 | 10 |  |  |
| *Calamagrostis arundinacea* | H | 1.1 | 10 | 10 | 20 | 50 |  |
| *Calamagrostis epigeios* | H | 2.1 | 70 | 30 | 10 |  |  |
| *Calluna vulgaris* | H | 2.1 | 40 |  |  |  |  |
| *Carex leporina* | H | 2.2 | 10 |  |  |  |  |
| *Carex nigra* | H | 2.1 | 10 |  |  |  |  |
| *Carex pilulifera* | H | 2.1 | 70 | 30 | 30 | 10 |  |
| *Dryopteris carthusiana* | H | 2.1/AFS |  | 10 | 20 | 60 | 10 |
| *Fagus sylvatica* | H |  |  |  |  | 10 |  |
| *Fragaria vesca* | H | 2.1 |  | 10 |  |  |  |
| *Frangula alnus* | H |  |  | 20 |  | 10 |  |
| *Juncus effusus* | H | 2.1 | 30 |  |  |  |  |
| *Luzula multiflora* | H | 2.1 | 10 |  |  |  |  |
| *Luzula pilosa* | H | 1.1/AFS | 10 | 10 | 10 | 10 |  |
| *Maianthemum bifolium* | H | 1.1/AFS | 10 |  | 10 | 10 |  |
| *Melampyrum pratense* | H | 1.1/AFS | 10 |  |  | 30 | 30 |
| *Moehringia trinervia* | H | 1.1 |  |  |  | 10 |  |
| *Molinia caerulea* | H | 2.1 | 10 |  |  |  | 20 |
| *Oxalis acetosella* | H | 1.1/AFS |  |  |  | 20 |  |
| *Pinus sylvestris* | H |  | 40 | 30 | 50 | 50 | 20 |
| *Populus tremula* | H |  | 10 |  |  |  |  |
| *Prunus serotina* | H |  |  | 20 |  |  |  |
| *Pteridium aquilinum* | H | 1.1/AFS |  | 10 | 10 | 10 | 20 |
| *Quercus petraea* | H |  | 60 | 60 | 60 | 80 | 70 |
| *Quercus robur* | H |  |  | 10 | 10 |  | 10 |
| *Quercus rubra* | H |  |  | 10 | 10 |  | 10 |
| *Rubus* sp. | H |  | 50 | 10 | 10 | 20 | 10 |
| *Rumex acetosella* | H | 2.2 | 10 |  |  |  |  |
| *Solidago gigantea* | H | 2.2 | 10 |  |  |  |  |
| *Sorbus aucuparia* | H |  | 10 |  | 30 | 10 |  |
| *Trientalis europaea* | H | 1.1 |  |  |  | 10 |  |
| *Vaccinium myrtillus* | H | 2.1/AFS | 100 | 100 | 100 | 100 | 100 |
| *Vaccinium vitis-idaea* | H | 2.1 | 40 | 40 | 70 | 60 | 60 |
| *Atrichum undulatum* | M | 2.1 | 10 |  |  |  |  |
| *Aulacomnium androgynum* | M | 1.2 | 10 | 10 |  |  |  |
| *Aulacomnium palustre* | M | 2.1 |  | 20 |  |  |  |
| *Brachythecium rutabulum* | M | 2.1 |  | 20 | 40 | 40 | 50 |
| *Brachythecium starkei* | M | 1.1 |  | 10 |  |  |  |
| *Campylopus introflexus* | M | 2.2 | 40 |  |  |  |  |
| *Campylopus pyriformis* | M | 2.2 | 10 |  |  |  |  |
| *Cephaloziella* sp. | M |  |  | 10 |  |  |  |
| *Ceratodon purpuraeus* | M | 2.2 | 70 |  |  |  |  |
| *Cladonia chlorophaea* | M | 2.1 | 30 |  |  |  |  |
| *Cladonia coniocraea* | M | 2.1 |  | 30 |  | 10 |  |
| *Cladonia fimbriata* | M | 2.1 | 10 |  |  |  |  |
| *Cladonia macilenta* | M | 2.1 | 40 |  |  |  |  |
| *Cladonia ochrochlora* | M |  |  | 20 |  |  |  |
| *Cladonia* sp. | M |  | 20 |  |  |  | 10 |
| *Coenogonium pineti* | M | 1.1 |  | 10 |  |  |  |
| *Dicranella heteromalla* | M | 2.1 | 80 | 10 | 20 |  | 20 |
| *Dicranum polysetum* | M | 2.1 | 50 | 70 | 40 | 20 | 50 |
| *Dicranum scoparium* | M | 2.1 | 40 | 50 | 30 | 10 | 20 |
| *Dicranum spurium* | M | 2.1 | 20 | 10 |  |  |  |
| *Herzogiella seligeri* | M | 1.1 |  | 10 |  | 20 | 10 |
| *Hylocomium splendens* | M | 2.1 | 10 |  | 10 | 10 | 60 |
| *Hypnum cupressiforme* | M | 2.1 | 10 | 10 |  | 20 |  |
| *Hypnum jutlandicum* | M | 2.1 | 60 | 40 | 30 | 30 | 40 |
| *Leucobryum glaucum* | M | 2.1 |  |  |  | 10 |  |
| *Lophocolea heterophylla* | M | 1.1 |  | 40 | 20 | 20 | 30 |
| *Micarea micrococca* | M | 2.1 |  | 10 |  |  |  |
| *Orthodicranum flagellare* | M | 1.1 | 10 |  |  |  |  |
| *Orthodicranum montanum* | M | 2.1 | 60 | 40 | 30 | 40 | 30 |
| *Orthodicranum tauricum* | M | 1.1 | 10 |  |  |  | 10 |
| *Orthodontium lineare* | M | 2.1 | 10 |  |  |  |  |
| *Placynthiella dasaea* | M | 2.1 | 10 | 10 | 10 |  | 30 |
| *Placynthiella icmalea* | M | 2.1 | 90 |  |  |  | 10 |
| *Placynthiella oligotropha* | M | 2.2 | 90 |  |  |  |  |
| *Plagiomnium affine* | M | 2.1 |  |  | 20 | 10 |  |
| *Plagiothecium curvifolium* | M | 2.1 |  | 40 | 40 | 50 | 50 |
| *Plagiothecium denticulatum* | M | 2.1 |  |  | 10 |  |  |
| *Plagiothecium laetum* | M | 2.1 |  |  |  |  | 10 |
| *Pleurozium schreberi* | M | 2.1 | 80 | 100 | 100 | 100 | 100 |
| *Pohlia nutans* | M | 2.1 | 100 | 20 |  | 20 | 50 |
| *Polytrichastrum formosum* | M | 2.1 | 80 | 20 | 50 | 20 | 60 |
| *Polytrichum juniperinum* | M | 2.2 | 50 | 10 |  |  |  |
| *Pseudoscleropodium purum* | M | 2.1 | 10 | 70 | 80 | 50 | 80 |
| *Ptilium crista-castrensis* | M | 1.1 |  |  |  |  | 20 |
| *Rhytidiadelphus squarrosus* | M | 2.2 |  |  |  | 10 |  |
| *Sciuro-hypnum oedipodium* | M | 2.1 | 10 | 80 | 80 | 60 | 80 |
| *Tetraphis pellucida* | M | 2.1 | 10 |  |  |  | 10 |
| *Trapeliopsis gelatinosa* | M | 2.1 |  | 10 |  |  |  |
| *Trapeliopsis granulosa* | M | 2.1 | 10 |  |  |  | 10 |
| *Trapeliopsis pseudogranulosa* | M | 2.1 |  |  |  | 10 |  |
| *Aulacomnium androgynum* | CWD | 1.2 | 10 | 20 |  |  |  |
| *Aulacomnium palustre* | CWD | 2.1 |  | 10 |  |  |  |
| *Brachythecium rutabulum* | CWD | 2.1 | 20 | 60 | 30 | 20 | 40 |
| *Brachythecium salebrosum* | CWD | 2.1 |  | 10 |  |  | 10 |
| *Brachythecium* sp. | CWD |  | 10 |  |  |  |  |
| *Brachythecium velutinum* | CWD | 2.1 |  |  |  | 10 |  |
| *Campylopus flexuosus* | CWD | 2.1 | 10 |  |  |  |  |
| *Cephalozia lunulifolia* | CWD | 1.1 | 10 |  |  |  |  |
| *Cephaloziella divaricata* | CWD | 2.2 | 10 |  | 10 |  |  |
| *Ceratodon purpuraeus* | CWD | 2.2 | 20 | 20 |  |  |  |
| *Cladonia chlorophaea* | CWD | 2.1 | 10 | 20 |  |  |  |
| *Cladonia coniocraea* | CWD | 2.1 | 70 | 60 | 50 | 40 | 30 |
| *Cladonia digitata* | CWD | 2.1 |  | 10 |  | 10 | 10 |
| *Cladonia fimbriata* | CWD | 2.1 |  | 10 |  |  |  |
| *Cladonia macilenta* | CWD | 2.1 | 40 | 20 | 10 |  |  |
| *Cladonia ochrochlora* | CWD |  |  | 10 |  |  |  |
| *Cladonia polydactyla* | CWD | 1.2 | 20 |  |  |  |  |
| *Cladonia* sp. | CWD |  | 20 | 20 | 30 | 10 | 20 |
| *Coenogonium pineti* | CWD | 1.1 |  | 60 | 10 | 20 | 40 |
| *Dicranella heteromalla* | CWD | 2.1 | 20 | 20 |  |  |  |
| *Dicranum polysetum* | CWD | 2.1 |  | 10 |  |  |  |
| *Dicranum scoparium* | CWD | 2.1 | 10 | 30 | 30 |  | 20 |
| *Herzogiella seligeri* | CWD | 1.1 |  | 70 | 40 | 20 | 20 |
| *Hypnum cupressiforme* | CWD | 2.1 | 10 | 70 | 10 | 30 | 40 |
| *Hypnum cupressiforme var. filiforme* | CWD |  |  |  |  |  | 10 |
| *Hypnum jutlandicum* | CWD | 2.1 | 10 | 40 |  | 20 | 20 |
| *Hypocenomyce scalaris* | CWD | 2.1 | 20 |  |  |  |  |
| *Hypogymnia physodes* | CWD | 2.1 | 10 | 10 |  |  | 10 |
| *Lepidozia reptans* | CWD | 1.1 |  |  |  | 10 |  |
| *Lepraria* sp. | CWD |  |  | 10 | 10 | 20 | 10 |
| *Lophocolea bidentata* | CWD | 2.1 |  | 10 |  |  |  |
| *Lophocolea heterophylla* | CWD | 1.1 | 10 | 90 | 80 | 70 | 60 |
| *Micarea botryoides* | CWD | 1.1 | 20 | 10 |  |  |  |
| *Micarea micrococca* | CWD | 2.1 | 10 | 20 | 10 |  | 20 |
| *Micarea misella* | CWD | 1.1 | 40 | 10 |  | 10 |  |
| *Orthodicranum montanum* | CWD | 2.1 | 50 | 80 | 70 | 40 | 60 |
| *Orthodicranum tauricum* | CWD | 1.1 |  | 10 |  |  |  |
| *Orthodontium lineare* | CWD | 2.1 |  | 30 |  |  | 10 |
| *Orthotrichum* sp. | CWD |  |  |  |  |  | 10 |
| *Peltigera collina* | CWD |  | 10 |  |  |  |  |
| *Placynthiella dasaea* | CWD | 2.1 | 40 | 80 | 40 | 10 | 70 |
| *Placynthiella icmalea* | CWD | 2.1 | 80 | 20 |  | 20 | 20 |
| *Placynthiella oligotropha* | CWD | 2.2 | 20 |  |  |  |  |
| *Plagiomnium affine* | CWD | 2.1 |  |  | 10 |  |  |
| *Plagiomnium cuspidatum* | CWD | 2.1 |  | 10 |  |  |  |
| *Plagiothecium curvifolium* | CWD | 2.1 |  | 50 | 30 | 20 | 30 |
| *Plagiothecium laetum* | CWD | 2.1 |  | 20 |  | 10 | 20 |
| *Plagiothecium* sp. | CWD |  |  |  | 10 |  |  |
| *Pleurozium schreberi* | CWD | 2.1 | 40 | 30 | 60 | 80 | 60 |
| *Pohlia nutans* | CWD | 2.1 | 70 | 60 | 40 | 50 | 10 |
| *Polytrichastrum formosum* | CWD | 2.1 | 10 | 10 |  |  | 10 |
| *Polytrichum juniperinum* | CWD | 2.2 |  | 10 |  |  |  |
| *Pseudoscleropodium purum* | CWD | 2.1 |  | 30 | 30 | 10 | 20 |
| *Rosulabryum moravicum* | CWD |  |  | 10 |  |  |  |
| *Sciuro-hypnum oedipodium* | CWD | 2.1 |  | 70 | 60 | 10 | 30 |
| *Scoliciosporum chlorococcum* | CWD | 2.2 |  | 10 |  |  |  |
| *Tetraphis pellucida* | CWD | 2.1 | 20 |  | 10 | 30 | 10 |
| *Trapeliopsis flexuosa* | CWD | 2.2 | 40 |  |  |  |  |
| *Trapeliopsis granulosa* | CWD | 2.1 | 30 |  |  |  | 20 |
| *Trapeliopsis pseudogranulosa* | CWD | 2.1 | 20 | 10 |  |  |  |
| *Brachythecium rutabulum* | Pinus | 2.1 |  |  | 10 |  |  |
| *Chaenotheca ferruginea* | Pinus | 1.1 |  |  |  |  | 30 |
| *Cladonia chlorophaea* | Pinus | 2.1 |  | 40 |  |  | 10 |
| *Cladonia coniocraea* | Pinus | 2.1 |  | 60 | 30 | 10 | 20 |
| *Cladonia digitata* | Pinus | 2.1 |  |  | 20 | 70 | 10 |
| *Cladonia fimbriata* | Pinus | 2.1 |  | 10 |  |  |  |
| *Cladonia macilenta* | Pinus | 2.1 |  | 10 | 30 | 40 |  |
| *Cladonia ochrochlora* | Pinus |  |  | 30 | 20 | 20 |  |
| *Cladonia* sp. | Pinus |  |  | 50 | 10 | 10 | 40 |
| *Coenogonium pineti* | Pinus | 1.1 |  | 100 | 40 | 30 | 10 |
| *Dicranum scoparium* | Pinus | 2.1 |  | 10 |  | 10 | 10 |
| *Herzogiella seligeri* | Pinus | 1.1 |  | 10 |  |  |  |
| *Hypnum cupressiforme* | Pinus | 2.1 |  | 10 | 10 |  |  |
| *Hypnum jutlandicum* | Pinus | 2.1 |  | 30 |  |  |  |
| *Hypocenomyce scalaris* | Pinus | 2.1 |  | 10 | 50 | 60 | 60 |
| *Hypogymnia physodes* | Pinus | 2.1 |  |  |  | 20 | 40 |
| *Lecanora conizaeoides* | Pinus | 2.2 |  | 10 | 50 | 10 | 10 |
| *Lecanora* sp. | Pinus |  |  |  | 10 |  |  |
| *Lepraria* sp. | Pinus |  |  | 30 | 70 | 50 | 10 |
| *Lophocolea heterophylla* | Pinus | 1.1 |  | 100 | 70 | 50 | 40 |
| *Micarea micrococca* | Pinus | 2.1 |  | 80 | 30 | 30 | 20 |
| *Micarea prasina* | Pinus | 1.1 |  | 10 | 20 | 10 |  |
| *Orthodicranum montanum* | Pinus | 2.1 |  | 80 | 40 | 20 | 30 |
| *Orthodontium lineare* | Pinus | 2.1 |  |  | 10 |  | 10 |
| *Placynthiella dasaea* | Pinus | 2.1 |  | 40 |  | 20 | 10 |
| *Plagiothecium curvifolium* | Pinus | 2.1 |  | 40 | 10 | 10 | 10 |
| *Plagiothecium laetum* | Pinus | 2.1 |  | 20 |  |  |  |
| *Pleurozium schreberi* | Pinus | 2.1 |  | 10 |  | 20 | 10 |
| *Pohlia nutans* | Pinus | 2.1 |  | 30 | 10 |  | 10 |
| *Sciuro-hypnum oedipodium* | Pinus | 2.1 |  | 20 |  | 10 |  |
| *Tetraphis pellucida* | Pinus | 2.1 |  |  |  |  | 20 |
| *Trapeliopsis flexuosa* | Pinus | 2.2 |  | 20 | 10 |  |  |
| *Trapeliopsis granulosa* | Pinus | 2.1 |  |  | 10 |  |  |
| *Aulacomnium androgynum* | Quercus | 1.2 |  |  |  |  | 10 |
| *Brachythecium rutabulum* | Quercus | 2.1 |  |  |  |  | 20 |
| *Brachythecium velutinum* | Quercus | 2.1 |  |  |  |  | 10 |
| *Cladonia* sp. | Quercus |  |  |  | 10 |  | 30 |
| *Coenogonium pineti* | Quercus | 1.1 |  | 20 |  | 10 | 30 |
| *Herzogiella seligeri* | Quercus | 1.1 |  |  |  |  | 10 |
| *Hypnum cupressiforme* | Quercus | 2.1 |  | 10 | 10 | 10 | 40 |
| *Hypnum cupressiforme var. filiforme* | Quercus |  |  |  |  |  | 20 |
| *Hypogymnia physodes* | Quercus | 2.1 |  |  |  |  | 10 |
| *Lepraria* sp. | Quercus |  |  |  | 20 |  | 30 |
| *Lophocolea heterophylla* | Quercus | 1.1 |  | 10 | 10 |  | 50 |
| *Micarea prasina* | Quercus | 1.1 |  |  |  |  | 10 |
| *Orthodicranum montanum* | Quercus | 2.1 |  | 10 | 10 |  | 10 |
| *Placynthiella dasaea* | Quercus | 2.1 |  |  |  |  | 10 |
| *Plagiothecium curvifolium* | Quercus | 2.1 |  |  | 20 | 10 | 30 |
| *Plagiothecium denticulatum* | Quercus | 2.1 |  |  |  |  | 10 |
| *Plagiothecium laetum* | Quercus | 2.1 |  |  | 10 | 10 | 30 |
| *Pleurozium schreberi* | Quercus | 2.1 |  |  |  |  | 20 |
| *Pohlia nutans* | Quercus | 2.1 |  |  | 10 |  | 30 |
| *Sciuro-hypnum oedipodium* | Quercus | 2.1 |  |  |  |  | 20 |
| *Tetraphis pellucida* | Quercus | 2.1 |  |  |  |  | 10 |
| *Hypnum jutlandicum* | Picea | 2.1 |  |  |  | 10 | 10 |
| *Lepraria* sp. | Picea |  |  |  |  | 20 | 10 |
| *Lophocolea heterophylla* | Picea | 1.1 |  |  |  | 20 |  |
| *Micarea prasina* | Picea | 1.1 |  |  |  | 10 |  |
| *Orthodicranum montanum* | Picea | 2.1 |  |  |  | 10 |  |
| *Plagiothecium curvifolium* | Picea | 2.1 |  |  |  | 10 | 20 |
| *Plagiothecium laetum* | Picea | 2.1 |  |  |  | 10 | 10 |
| *Pohlia nutans* | Picea | 2.1 |  |  |  |  | 10 |
| *Herzogiella seligeri* | Betula | 1.1 |  |  |  |  | 10 |
| *Hypnum cupressiforme* | Betula | 2.1 |  |  |  |  | 10 |
| *Lophocolea heterophylla* | Betula | 1.1 |  |  |  |  | 10 |
| *Orthodicranum montanum* | Betula | 2.1 |  |  |  |  | 10 |
| *Plagiothecium curvifolium* | Betula | 2.1 |  |  |  |  | 10 |
| *Sciuro-hypnum oedipodium* | Betula | 2.1 |  |  |  |  | 10 |
| *Tetraphis pellucida* | Betula | 2.1 |  |  |  |  | 10 |

Stand age classes: 1 – initiation stands (4–10 years), 2 – young stands (20–35 years), 3 – middle-aged stands (45–60 years), 4 – pre-mature stands (70–85 years), 5 – mature stands (95–110 years); layers/substrates: T1 – high tree layer, T2 – low tree layer, S1 – high shrub layer, S2 – low shrub layer, H – herb layer, M – bryophyte-lichen layer, CWD – coarse woody debris, Pinus, Quercus, Picea – trunks as substrates for epiphytes; species category: 1.1 – species restricted to closed forests, 1.2 – species preferring forest edges and clearings, 2.1 – species occurring in forests and in open land, 2.2 – species occurring in forests, but preferring open land, AFS – ancient forest species.
